# CRISPR/Cas9 delivery with one single adenoviral vector devoid of all viral genes

**DOI:** 10.1038/s41598-017-17180-w

**Published:** 2017-12-07

**Authors:** Eric Ehrke-Schulz, Maren Schiwon, Theo Leitner, Stephan Dávid, Thorsten Bergmann, Jing Liu, Anja Ehrhardt

**Affiliations:** Institute of Virology and Microbiology, Center for Biomedical Education and Research (ZBAF), Department of Human Medicine, Faculty of Health, Witten/Herdecke University, Witten, Germany

## Abstract

The Clustered Regularly Interspaced Short Palindromic Repeats (CRISPR)/Cas9 system revolutionized the field of gene editing but viral delivery of the CRISPR/Cas9 system has not been fully explored. Here we adapted clinically relevant high-capacity adenoviral vectors (HCAdV) devoid of all viral genes for the delivery of the CRISPR/Cas9 machinery using a single viral vector. We present a platform enabling fast transfer of the Cas9 gene and gRNA expression units into the HCAdV genome including the option to choose between constitutive or inducible Cas9 expression and gRNA multiplexing. Efficacy and versatility of this pipeline was exemplified by producing different CRISPR/Cas9-HCAdV targeting the human papillomavirus (HPV) 18 oncogene *E6*, the dystrophin gene causing Duchenne muscular dystrophy (DMD) and the HIV co-receptor C-C chemokine receptor type 5 (CCR5). All CRISPR/Cas9-HCAdV proved to be efficient to deliver the respective CRISPR/Cas9 expression units and to introduce the desired DNA double strand breaks at their intended target sites in immortalized and primary cells.

## Introduction

The CRISPR/Cas9 system is widely used for various genome editing approaches in cultured cells and living organisms and was broadly explored for preclinical applications. This two component system is composed of the Cas9 endonuclease that acts in cooperation with a chimeric guide RNA (gRNA) mediating the sequence-specific binding to its complementary target protospacer sequence preceding a protospacer adjunct motif (PAM)^1^. Due to its simple gRNA design and easy cloning procedure for customization, the CRISPR/Cas9 system is easier to handle than transcription activator-like effector nucleases (TALENs) and artificial zinc finger nucleases (ZFN)^2^. Moreover the relatively small size of its coding sequence enables the introduction of the Cas9 encoding DNA into retroviral, lentiviral, and adeno-associated viral (AAV) vectors, which have been used as CRISPR/Cas9 delivery tools^3–7^. Studies utilizing adenoviral (AdV) vectors as delivery vehicles for CRISPR/Cas9 showed efficient gene disruption in the host genome of various human cells^8^ and in viral genomes^9^ in antiviral approaches. Furthermore, AdV-mediated delivery was utilized to disrupt the HIV co-receptor in T-cells^10^, to perform genome editing in adult murine liver^11^ and for restoration of the dystrophin gene *in vitro* ^12^ and *in vivo* ^13^. However, all of the latter studies applied early generation AdV vectors based on the human AdV type 5 deleted for the adenoviral early genes E1 and E3, which are known to cause anti-adenoviral innate and adoptive immune responses^14^ suppressing effects of the viral vector. Importantly, the commonly used human AdV type 5 elicits formation of CD4 and CD8 positive T-lymphocytes^15,16^ and pre-existing immunity of up to 90% in the human population can be detected^17^. This can be partially attributed to leaky expression of remaining adenoviral genes in the recombinant AdV genome.

No attempts have been made to deliver CRISPR/Cas9 using newest generation high-capacity adenoviral vectors (HCAdV)^18^, even though they provide various advantages. As they are gene-deleted, they do not express any viral genes leading to less immunogenicity of the viral vector^19,20^. In contrast to retroviral- and lentiviral vectors which integrate into the host genome and AAV vectors which were also shown to partially integrate their genetic payload into host chromosomes, AdV predominantly persist extrachromosomally providing an improved genotoxicity profile^21^. Furthermore the packaging capacity of up to 35 kb of HCAdV allows to incorporate further genes such as fluorescent or luminescent reporters or to use the inducible TetOn3G system that would need an additional transactivator gene. Adaption of the HCAdV vector system for CRISPR/Cas9 delivery was generally hampered by the complicated handling of large constructs during cloning and the work and time intensive procedure of vector production. To overcome these limitations novel means are needed that facilitate fast construction of HCAdV genomes containing all components of the CRISPR/Cas9 system. For this purpose we generated intermediate shuttle plasmids for fast cloning of customized gRNAs and subsequent insertion of all CRISPR/Cas9 components into the HCAdV genome either contained in a bacterial artificial chromosome (BAC)^22^ or the established pAdFTC plasmid^20^.

Production of viral vectors carrying all components of the designer nuclease machinery in a single vector may be challenging because this may result in unwanted side effects due to constant induction of DNA double-strand breaks (DSB) within the genome of the producer cells. To produce HCAdVs containing expression cassettes for a complete TALEN pair, a previous study introduced endogenous micro RNA regulated transgene expression to avoid TALEN expression in HCAdV producer cells during vector amplification^23^. In our vector pipeline we included two versions of intermediate plasmids for either constitutive Cas9 expression via the truncated hybrid chicken beta actin promotor (CBh) promotor^24^ or inducible expression using the TetOn3G system^25,26^. Furthermore the intermediate shuttle plasmids allow the introduction of several gRNAs for multiplexing. Here we evaluated this vector pipeline for delivery of gRNAs targeting clinically relevant targets including disruption of the HPV 18 genome, the HIV co-receptor CCR5, and the dystrophin gene affected in patients suffering from DMD.

## Results

### Construction of intermediate CRISPR-/Cas9 shuttle plasmids and generation of HCAdV vector genomes by recombineering or endonuclease guided cloning

**Figure 1:**
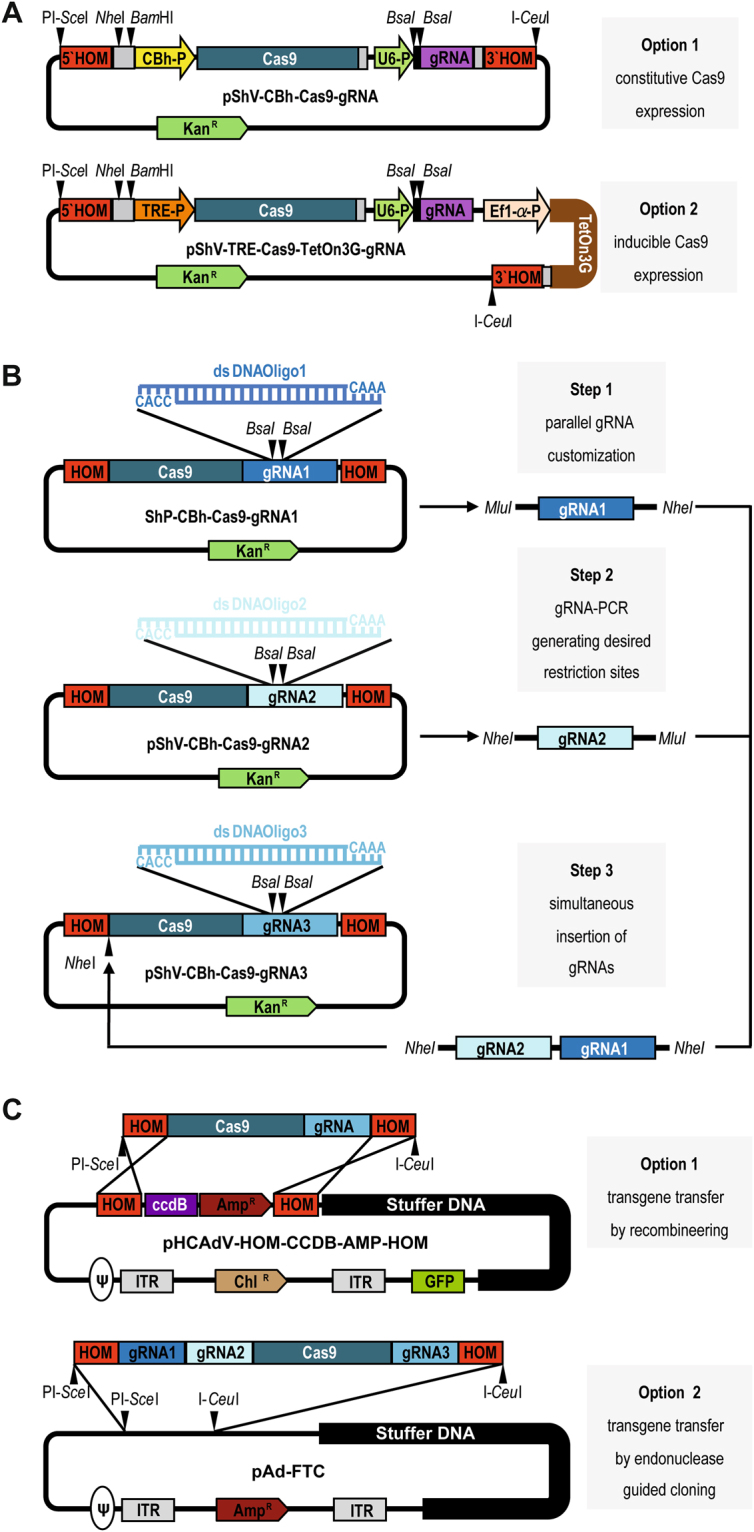
Plasmid toolbox for the construction of CRISPR/Cas9-HCAdV genomes. (**A**) Schematic presentation of intermediate CRISPR/Cas9 shuttle plasmids for simple gRNA manipulation and multiplexing and subsequent transfer of the customized CRISPR/Cas9 machinery into the HCAdV genome. Option 1: pShV-CBh-Cas9-gRNA for constitutive Cas9 expression. Option 2: pShV-TRE-Cas9-TeOn3G-gRNA for inducible Cas9 expression utilizing the TetOn3G system. Black arrowheads indicate unique restriction enzyme sites for insertion of further gRNA expression units. (**B**) Workflow for gRNA customization and multiplexing of the CRISPR/Cas9 machinery. Step1: Complementary annealed gRNA oligonucleotides are separately inserted between the *Bsa*I restriction enzyme sites resulting in pShV-CBh-Cas9-gRNA1, pShV-CBh-Cas9-gRNA2 and pShV-CBh-Cas9- gRNA3. Step 2: Customized gRNA expression units gRNA1 and gRNA2 are amplified by PCR using primers generating desired restriction enzyme sites. Step 3: gRNA1 and 2 are inserted into the respective restriction enzyme site within pShV-CBh-Cas9-gRNA1 resulting in pShV-CBh-Cas9-CBh-gRNA1-gRNA2-gRNA3. (**C**) Transfer of customized CRISPR/Cas9 transgenes into the HCAdV genomes. Option 1: Released CRISPR/Cas9 transgene cassettes flanked by homology arms are inserted into pHCAdV-HOM-CcdB-AMP-HOM replacing the CcdB-Amp^R^ cassette. Option 2: Endonuclease guided cloning into pAd-FTC utilizing PI-*Sce*I and I-*Ceu*I. HOM, homology arms for homologous recombination into pHCAdV-HOM-CCBD-AMP-HOM; CBh-P, constitutive hybrid CMV enhancer/chicken β-actin promotor; TRE-P, inducible tetracycline responsible element promotor; TetOn3G, TetOn3G transactivator; Ef1-α-P, Ef1-α-Promotor; Cas9, Streptococcus pyogenes Cas9, gRNA, guide RNA expression unit; U6-P, U6 RNA polymerase III promotor, Kan^R^, Kanamycin resistance cassette; Amp^R^; Ampicillin resistance cassette, Chl^R^, Chloramphenicol resistance cassette; CcdB, control of cell death B expression cassette; ITR, adenovirus serotype 5 inverted terminal repeat; Ψ, adenovirus serotype 5 packaging signal.

We present a fast pipeline to customize and clone or recombine all CRISPR/Cas9 components into the HCAdV genome, which is schematically outlined in Fig. 1. For simple customization of gRNAs and subsequent transfer of the CRISPR/Cas9 components into the HCAdV genome we constructed two versions of shuttle plasmids (pShV) containing a Cas9 gene and a customizable gRNA expression unit. pShV-CBh-Cas9-gRNA contains the Cas9 nuclease gene utilizing a constitutive hybrid CMV enhancer/chicken β-actin promotor (CBh)^24^. pShV-TRE-Cas9-TetOn3G-gRNA expresses Cas9 via the inducible TRE-promotor in combination with a TetOn3G inducer expressed under the control of the Ef1-α-promotor^25,26^ (Fig. 1A). Plasmid sequences are provided in the electronic supplementary material.

The gRNA expression unit in both pShV contain a short DNA sequence comprising of a *Spe*I restriction enzyme site flanked by two *Bsa*I restriction enzyme sites at the position that needs to be manipulated to specify the RNA guided Cas9 binding to the desired target DNA sequence. The guide RNA expression unit can be customized by simple insertion of a target specific dsDNA oligonucleotide having respective overhangs for the restriction enzyme *Bsa*I (Fig. 1B). To allow the use of multiple gRNA expression units for multiplexing of the CRISPR/Cas9 system, further gRNA expression units can be inserted into the shuttle plasmid. Additional gRNA expression units can be amplified via PCR using primers generating restriction enzyme sites for consecutive cloning of amplified gRNA expression units into respective restriction sites within pShV (Fig. 1B).

For efficient insertion of the CRISPR/Cas9 system including gRNA expression units by recombineering or endonuclease guided cloning, the shuttle vector pShV contains homology arms (HOM) flanking the CRISPR/Cas9 expression units enabling high throughput recombineering of the CRISPR/Cas9 components into the HCAdV genome contained in the BAC pBHCAdV-HOM-CCDB-AMP-HOM (sequence information are provided in the supporting material). Note that for efficient recombineering we used the selection marker CcdB^27,28^ enabling enhanced and improved recombineering. Alternatively, the homology arms are flanked by homing endonuclease restriction site for PI-*Sce*I and I-*Ceu*I, which allows transferring the CRISPR/Cas9 expression units into the HCAdV genome by homing endonuclease guided ligation into the PI-*Sce*I and I-*Ceu*I recognition sites present in the HCAdV contained in pAd-FTC^20^ (Fig. 1C).

### Applying the HCAdV pipeline for generation of recombinant HCAdV genomes

By applying the shuttle plasmids pShV-CBh-Cas9-gRNA and pShV-TRE-Cas9-TetOn3G-gRNA we created CRISPR expression constructs targeting different DNA sequences. gRNA sequences targeting these DNA sequences are summarized in Supplementary Table 1 and all oligonucleotides used for PCRs and cloning are listed in Supplementary Table 2. For the first construct carrying a single gRNA, the previously published DNA oligonucleotide t1^29^ specific for the HPV18 oncogene *E6* was introduced into pShV-CBh-Cas9-gRNA resulting in pShV-CBh-Cas9-gRNAHPV18E6 (Supplementary Figure 1). To prove our concept for inducible Cas9 expression, a gRNA specific for the HIV co-receptor CCR5 was generated and introduced into pShV-CBh-Cas9-gRNA as well as pShV-TRE-Cas9-Teton3G-gRNA, resulting in pShV-CBh-Cas9-gRNACCR5-88 and pShV-TRE-Cas9-TetOn3G-gRNACCR5-88, respectively (Supplementary Figure 2). To deliver two previously published gRNAs which are specific for intronic sequences flanking exon 51 of the human dystrophin gene^30^, oligonucleotides Cr1 and Cr5 were separately inserted into the gRNA expression units of pShV-CBh-Cas9-gRNA resulting in pShV-CBh-Cas9-gRNA-Cr5 and pShV-CBh-Cas9-gRNA-Cr1. Subsequently the gRNACr1 expression unit was introduced into pShV-CBh-Cas9-gRNA-Cr5 resulting in pShV-CBh-Cas9-gRNA-Cr1-Cr5 according to the scheme presented in Fig. 1B (Supplementary Figure 3). Functionality of cloned gRNAs targeting the the HPV18 oncogene E6 and the intronic sequences flanking exon 51 of the human dystrophin gene was shown before^29,30^. The gRNA CCR5-88 specifically targeting the CCR5 locus was generated in this study and functionality was shown after transfection of plasmid pX330-CBh-Cas9-gRNACCR5-88 into A549 cells (Supplementary Figure 4). On genomic level we observed efficiencies of up to 3.3%.

Finally we recombined the CRISPR/Cas9 expression cassettes from pShV-CBh-Cas9-gRNAHPV18E6 into pHCAdV-eGFP-HOM-CCBD-AMP-HOM resulting in pHCAdV-eGFP-CBh-Cas9-gRNAHPV18E6 (Supplementary Figure 1). The transgene cassettes of the other constructs (pShV-CBh-Cas9-gRNACCR5-88 and pShV-TRE-Cas9-TetOn3G-gRNACCR5-88, and pShV-CBh-Cas9-gRNACr1-Cr5) were inserted into the HCAdV genome by endonuclease guided cloning using an accelerated protocol when compared to the previously published protocol^31^. For details regarding this protocol please refer to the material and methods section. This resulted in pAd-FTC-CBh-Cas9-gRNACCR5-88, pAd-FTC-TRE-Cas9-TetOn3G-gRNACCR5-88 and pAd-FTC-CBh-Cas9-gRNACr1-Cr5 (Supplementary Figures 2 and 3).

### Vector amplification and purification

The HCAdV genome contained in pBHCA-eGFP-CBh-Cas9-gRNAHPV18E6 was released from the pBHCA backbone by AfeI and HpaI restriction digest (Supplementary Figure 1). HCAdV genomes contained in pAd-FTC (pAd-FTC-CBh-Cas9-gRNACCR5-88, pAd-FTC-TRE-Cas9-TetOn3G-gRNACCR5-88, and pAd-FTC-CBh-Cas9-gRNACr1-Cr5) were released from the pAd-FTC plasmid backbones by *Not*I restriction enzyme digest. Linearized HCAdV genomes were then transfected into 116 producer cell cells^32^ that were co-transduced with the adenovirus helper virus^32^ at the multiplicity of infection (MOI) 5. Vectors were amplified by a shortened protocol of consecutive passaging of cell-vector lysate and purified according to the scheme in Supplementary Figure 5. This modified vector amplification and purification procedure enables accelerated medium scale HCAdV production saving at least one week for each vector preparation. With this procedure we obtained sufficient vector titers for most CRISPR-HCAdV (Table 1). Only HCAdV-CBh-Cas9-CCR5gRNA-88 containing the Cas9 gene under control of the constitutive CBh-Promotor revealed low titers (Table 1). In contrast HCAdV-TRE-Cas9-TetOn3G-CCR5gRNA-88 using the inducible Tre promotor to control Cas9 expression was sufficiently amplified (Table 1). HCAdV-eGFP-CBh-Cas9-gRNAHPV18E6 could be amplified to the highest extent and yielded highest titers among all CRISPR-HCAdV, even though it also exploits the CBh-promotor to control Cas9 expression (Table 1). Interestingly, HCAdV-eGFP-CBh-Cas9-gRNAHPV18E6 that was released from the BAC-backbone by *Afe*I and *Mfe*I restriction enzyme digest, leaving a 3.4 kb fragment of BAC-backbone connected to the viral genome ends, also amplified sufficiently, suggesting that not precise release of the viral genome but rather linearization of the vector construct is essential for amplification of the HCAdV-genome within the producer cells. Also the double gRNA containing HCAdV-CBh-Cas9-gRNACr1-Cr5 targeting *DMD* showed sufficient amplification rates (Table 1).

**Table 1:** Vector titers of CRISPR-HCAdV preparations. TU, transducing units; vp, viral particles.

Vector	TU/ml	vp/ml
HCAdV-CBh-Cas9-gRNACCR5-88	7.2E + 06	6.4E + 11
HCAdV-TRE-Cas9-Teton3G-CCR5-gRNACCR5-88	2.5E + 08	7.5E + 11
HCAdV-eGFP-CBH-Cas9-gRNAHPV18E6	6.1E + 10	5.6E + 11
HCAdV-CBh-Cas9-gRNACr5+CR1	1.3E + 08	7.0E + 11

### Viral delivery and detection of CRISPR/Cas9 mediated double-strand DNA break induction

After successful amplification and purification of all vectors, immortalized and primary cells were transduced to show efficacy of genome editing rates. A549 cells were transduced with HCAdV-Tre-Cas9-Teton3G-gRNACCR5 targeting CCR5 and HCAdV-eGFP-CBh-Cas9-gRNAHPV18E6 targeting HPV was used to transduce Hela cells carrying HPV18 DNA integrated into the cellular genome. Primary human skeletal myoblasts cells were transduced with HCAdV-CBh-Cas9-gRNACr1-Cr5 targeting the dystrophin gene.

**Figure 2:**
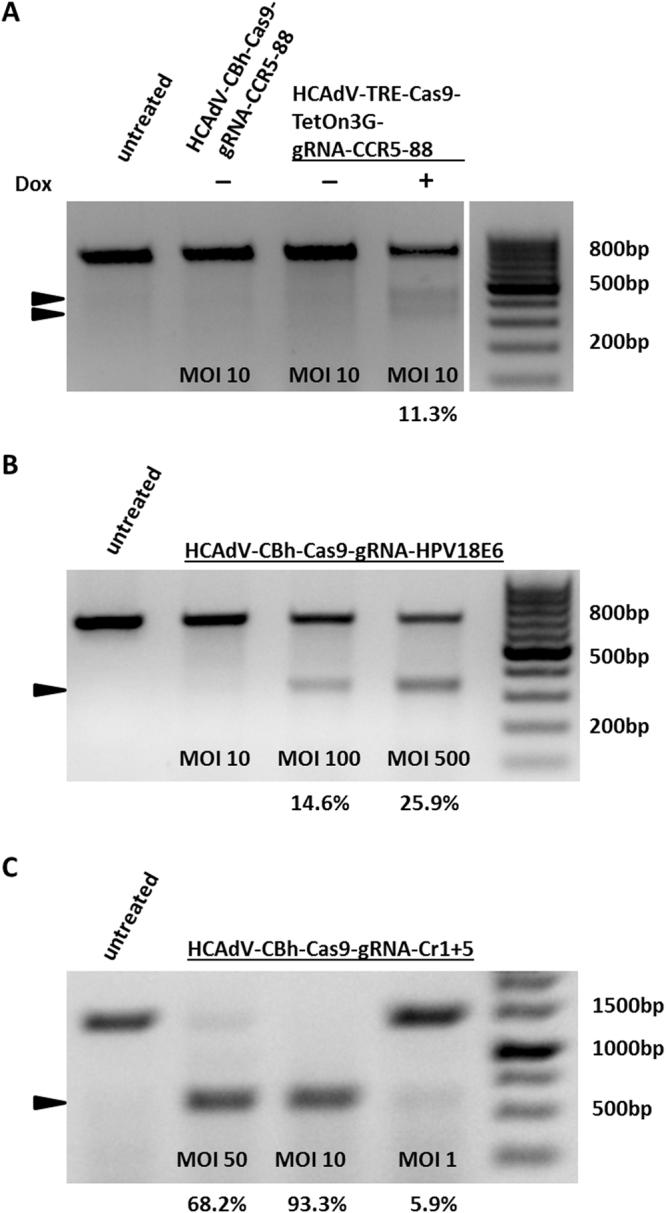
Functionality tests of CRISPR-HCAdV in A549, HeLa and primary human skeletal myoblasts. Cells were transduced with different multiplicities of infection (MOI). Two days post-transduction genomic DNA was extracted. (**A**) A549 cells were transduced with HCAdV-Cbh-Cas9-gRNA-CCR5-88 or HCAdV-TRE-Cas9-Teton3G-gRNA-CCR5-88 and for cells that were transduced with HCAdV-TRE-Cas9-Teton3G-gRNA-CCR5-88 Cas9, expression was induced by doxycycline. Displayed is an agarose gel after T7E1 digest of PCR products of the amplified CCR5 locus. Arrowheads indicate specific cleavage products. (**B**) Hela cell transduced with HCAdV-EGFP-CBh-Cas9-gRNAHPV18E6. The HPV18 *E6* locus was PCR-amplified and a T7E1 assay performed. Arrows show specific cleavage products (**C**) **P**rimary human skeletal myoblasts were transduced with HCAdV-CBh-Cas9-gRNACr1-Cr5. PCR products of amplified DMD locus revealed specific exon deletion. The border of the gels outside of the lanes was cropped. Note that the displayed gel in Fig. 2A was cropped from different parts of the gel and subsequently grouped. Activities are shown as percentages below the respective lanes.

T7E1 mutation detection assay showed substantial gene editing in genomic DNA extracted from A549 cells that were transduced with HCAdV-Tre-Cas9-Teton3G-gRNACCR5 and supplemented with doxycycline (Fig. 2A). We observed CRISPR/Cas9 activities of HCAdV-mediated gRNA delivery of 11.3% (Fig. 2A). T7E1 mutation detection assay also showed gene editing in gDNA extracted from Hela cells transduced with HCAdV-eGFP-CBh-Cas9-gRNAHPV18E6 with efficiencies of up to 25.9% (Fig. 2B). Deletion detection PCR showed successful deletion of dystrophin gene exon 51 after transduction of primary human skeletal myoblasts with highest CRISPR/Cas9 activities with up to 93.3% (Fig. 2C). This shows that HCAdV is efficient in delivering the CRISPR/Cas9 machinery to mediate single as well as multiplex gene editing by utilizing either constitutive or inducible Cas9 expression in immortalized and primary cells.

## Discussion

We present a versatile toolbox to customize and clone or recombine all CRISPR/Cas9 components including multiple gRNA expression units into the HCAdV genome and to produce HCAdV vectors for the delivery of all CRISPR/Cas9 components for gene editing approaches using only one single vector.

Although HCAdV offer substantial advantages over other viral vector systems handling and manipulation of large DNA constructs such as AdV genomes remains complicated. To overcome these limitations we designed our intermediate CRISPR-shuttle plasmids to contain homology arms enabling high-throughput recombineering. Furthermore restriction sites for PI-*Sce*I and I-*Ceu*I flanking the homology arms allow transferring the CRISPR/Cas9 expression units into the HCAdV genome by homing endonuclease guided ligation. With this we offer the possibility to manipulate the HCAdV genome constructs also for research laboratories in which the recombineering technology is not established. Another advantage of the latter cloning option is that repeated sequences shared by different expression cassettes such as gRNA might cause intramolecular recombination during the recombineering procedure. To prove the potential of our pipeline we created several HCAdV genomes containing the Cas9 gene together with one or two different gRNA expression units either utilizing constitutive Cas9 expression via the CBh promotor or inducible Cas9 expression via the TetOn system. We demonstrate transfer of the assembled CRISPR/Cas9 expression units into the HCAdV genome by either recombineering or endonuclease guided cloning. This offers the possibility to choose the optimal strategy for the desired outcome by combining the benefits of both concepts. Note that our accelerated procedure of endonuclease guided cloning when directly compared to our previously published strategy^31^ saves time. However, it is not as fast and not as well suited for high throughput purposes as the recombineering procedure.

We exemplified the potential of our production pipeline by amplifying and purifying a series of CRISPR/Cas9-HCAdVs containing gRNAs against different DNA targets, such as human *CCR5*, human *DMD* and *HPV18E6* using a protocol for production of medium scale vector preparations. This allows shortening the time and work intensive procedure by one week and is especially essential if a large number of different CRISPR-HCAdV need to be produced and characterized initially. Titers of final vector preparations were 10- to 100-fold lower as for large scale preparations as expected but still high enough to characterize vectors *in vitro*. Note, that if a large amount of vectors is required, for instance for *in vivo* applications, it is possible to change the vector amplification protocol allowing large-scale production of HCAdV^31,33^.

HCAdV-CBh-Cas9-gRNACr5-Cr1 targeting *DMD* and HCAdV-eGFP-CBh-Cas9-gRNAHPV18E6 targeting *HPV18E6* amplified sufficiently and yielded expected vector titers, but for HCAdV-CBh-Cas9-gRNACCR5-88 targeting *CCR5* utilizing constitutive Cas9 expression we observed poor vector amplification and low vector yield (Table 1). This is in concordance with the observation that designer nuclease expressing viral vectors show low amplification rates if all components of the system are expressed from the same construct as exemplified for the TALEN system^23^. However, miRNA-regulated expression that suppresses transgene expression in HCAdV producer cells improved vector amplification^23^. Another study also showed that amplification of recalcitrant HCAdV that do not amplify efficiently, can be increased to higher final vector titers by miRNA mediated downregulation of transgene expression during vector production^34^. Therefore we considered keeping the Cas9 expression low during vector production using the TetOn system to overcome amplification deficiencies of the CCR5 specific CRISPR/Cas9-HCAdV construct. We exchanged the constitutively active hybrid CMV enhancer/chicken β-actin promotor (CBh)^24^ controlling Cas9 expression with the inducible TRE-promotor in combination with a TetOn3G inducer expressed via the Ef1-α promotor^25,26^. The novel construct HCAdV-TRE-Cas9-TetOn3G-gRNACCR5-88 was then amplified in absence or the Doxycycline inducer to avoid Cas9 expression during vector production. This strategy improved vector amplification and lead to sufficient final vector titers (Table 1). To be able to choose between constitutive and inducible Cas9 expression we included this option to our pipeline to provide the optimal setup for vector production especially for vectors that poorly amplify. The reason for this difference in vector amplification rates for CRISPR/Cas9-HCAdV containing gRNAs for certain genes remains unclear. HCAdV-CBh-Cas9-gRNAHPV18E6, HCAdV-CBh-Cas9-gRNACCR5-88 and HCAdV-CBh-Cas9-gRNACr5-Cr1 only differ in the sequence and or number of gRNA expression units. We speculate that expression of the CRISPR/Cas9 system during vector amplification could lead to target cleavage within the producer cell line which may somehow negatively affect replication or packaging of HCAdV. Further investigations are needed to provide detailed insights into this phenomenon.

CRISPR/Cas9 is especially valuable for the research community as multiplexing can be achieved by simply expressing multiple gRNAs for different targets. Therefore we included this option in our CRISPR/Cas9-HCAdV production procedure. We would like to emphasize that there are also other technologies for cloning of multiple gRNA expression units such as the Golden Gate cloning method^35^, which would have been also suitable for the construction of HCAdV harboring multiplex CRISPR/Cas9-based genome engineering system. However, as this would have necessitated extensive changes within the shuttle vector pShV-CBh-Cas9-gRNA, we decided to keep the procedure simple by providing a method based on standard cloning techniques.

After initial insertion of the gRNA oligonucleotide into the gRNA expression unit of pShV-CBh-Cas9 the gRNA expression unit can be amplified using primers that produce an overhang containing a restriction site present in pShV-CBh-Cas9-gRNA that has been customized with a different gRNA oligo before. By choosing primers that produce overhangs containing different restriction enzyme sites, a simultaneous integration of several additional gRNAs can be achieved in one cloning step (Fig. 1B). The possibility to multiplex the CRISPR/Cas9 system offers the potential to not only target one single locus within the target cells. For example it allows precise deletion of exons^30^ or the simultaneous targeting of several target sites within the genomes of viral pathogens^36,37^. Multiplex CRISPR approaches have been successfully used for treatment of Duchenne muscular dystrophy in a MDX mouse model^38,39^ utilizing adeno-associated virus (AAV) vectors for delivery. However, for AAV vectors the CRISPR/Cas9 components had to be co-delivered by two separate vectors, one carrying Cas9 and the other expressing the respective gRNA expression units. In contrast in our approach using one CRISPR/Cas9-HCAdV all guide RNAs and Cas9 can be delivered to the same target cell. Note that recently a Cas9 gene originating from Staphylococcus aureus (SaCas9) which is only 3.2 kb in size was introduced and its potential for gene editing approaches has been characterized^40^. Subsequently AAV vectors were generated to deliver all components of the CRISPR/SaCas9 system^41^ within one AAV overcoming the limitations of AAV packaging capacity. Therefore, both AAV vectors and HCAdV are promising tools for *in vivo* genome editing. Note that the packaging capacity of HCAdV not only allows delivering all CRISPR/Cas9 components but allows including for instance the TetOn system for inducible Cas9 expression that requires an additional expression cassette for the TetOn3G inducer gene. Apart from that CRISPR/Cas9-HCAdV offer the potential to include multiple gRNA expression units. Additional reporter genes such as *GFP* or luciferase can be included as well to be able to assess transfection efficiencies in cell culture or vector distribution upon *in vivo* administration.

Gene editing activities of the HCAdV-delivered CRISPR/Cas9 systems showed vector-dependent differences. Due to the low final vector titer the applied dose of the vector HCAdV-CBh-Cas9-gRNACCR5-88 could not be increased which may have led to measurable gene editing activities of the *CCR5* locus. Besides dose dependencies, another critical parameter for achieving optimal gene editing activities may be accessibility of the targeted locus including the chromatin structure. However, further studies need to be performed to address these issues in more detail.

All previous clinical studies utilizing adenoviral vectors used early generation adenoviral vectors that are only deleted for one or two early adenoviral genes. In contrast to HCAdV these early generation vectors cause strong innate and adoptive immune responses^14–17^ and therefore, we believe that a HCAdV is clearly superior and the vector of choice when considering clinical studies. This is supported by numerous *in vivo* experiments using HCAdVs^42,43^ and direct *in vivo* comparisons of early generation adenoviral vectors and HCAdV^19,20,44^, which demonstrated an improved safety and toxicity profile for HCAdV. Furthermore, in an unpublished *in vivo* study using HCAdVs expressing the CRISPR/Cas9 system for antiviral treatment, we observed no unwanted side effects after performing limited toxicity studies. We believe that after the fatal clinical trial in 1999 in which a patient suffering from ornithine transcarbamylase deficiency died after systemic administration of an early generation adenoviral vector^45,46^, this type of vector is unlikely to be approved for clinical trials in the future; especially for treatment of non-cancerous diseases.

In summary our vector pipeline can be applied for customization and multiplexing of the CRISPR/Cas9 system. It allows choosing between constitutive and inducible Cas9 expression and facilitates subsequent transfer of all components of the customized CRISPR/Cas9 system into the HCAdV genome by recombineering or endonuclease guided cloning. We demonstrate that HCAdV mediated gene transfer results in substantial CRISPR/Cas9 mediated gene editing for various clinically relevant target genes in immortalized and primary cells. Therefore, our vector pipeline may pave the way towards preclinical and clinical application of HCAdV.

## Methods

### Construction of intermediate CRISPR/Cas9 shuttle plasmids for subsequent cloning or recombineering into HCAdV genomes

The intermediate shuttle plasmid was constructed by insertion of a synthetic DNA fragment into the PexK plasmid (Eurofins). The synthetic DNA fragment is composed of a multiple cloning site (MCS) that is flanked by 112 bp and 116 bp non coding random DNA sequences, that do not share similarity with natural DNA sequences functioning as homology arms (HA) for site directed homologous recombination (HR) of any insert into HCAdV genomes contained in a bacterial artificial chromosome (BAC)^22^. The homology arms flanked MCS is surrounded by the recognition sites for the homing endonucleases I-*Ceu*I and PI-*Sce*I enabling the cloning of any insert into the respective restriction sites in the HCAdV genome contained in the previously established plasmid pAdFTC^20^. Outside of the I-*Ceu*I and PI-*Sce*I restriction sites we added recognition sites for the restriction enzyme *Swa*I to release the synthetic DNA fragment from the PexK plasmid. The plasmid backbone was digested with *Mfe*I and *Eco*RI to remove the original MCS of PexK, blunted and ligated with the released synthetic DNA. The resulting shuttle plasmid (pShV) served as the master plasmid to create intermediate shuttle plasmids for CRISPR/Cas9 cloning/recombineering into the HCAdV genome.

The Cas9 expression cassette driven by the CBh promotor and its bovine growth hormone polyadenylation site (pA) was released from the original plasmid pX330-U6-Chimeric_BB-CBh-hSpCas9 (Addgene) by *Not*I and *Xba*I digest, blunted and inserted into the *Eco*RV restriction enzyme site in the MCS of the pShV resulting in the plasmid pShV-CBh-Cas9. For construction of a CRISPR/Cas9 shuttle plasmid utilizing the TRE-promotor for inducible Cas9 expression, the TRE promotor was released from the pTRE3G-Ires plasmid (Clontech Laboratories, France) via *Taq*I restriction digest and inserted into the *Acc*I restriction site of the pShV resulting in the plasmid pShV-TRE. Subsequently the Cas9 coding sequence including polyadenylation signal (pA) was amplified by PCR from plasmid pX330-U6-Chimeric_BB-CBh-hSpCas9 using primers Cas9_fwd_AccI and Cas9_rev_XmaI generating restriction sites for AccI and XmaI. The amplified PCR product was then digested with *Acc*I and *Xma*I and inserted downstream of TRE promotor of pShV-TRE resulting in the plasmid pShV-TRE-Cas9. After that the Tet3G inducer expression cassette with its EF-1α promotor and SV40pA was amplified by PCR from the plasmid pEF-1alpha-Tet3G (Clontech Laboratories) using primers Tet3G_PacI_fwd and Tet3G_PacI_rev generating restriction sites for *Pac*I. The amplified PCR product was digested with *Pac*I and ligated into pShV-TRE-Cas9 downstream of the Cas9 gene resulting in plasmid pShV-TRE-Cas9-TetOn3G. The complete sequence of both shuttle plasmids is available in the Supplementary information.

We modified the gRNA expression cassette from pX330 by inserting an oligonucleotide (5′ CACCGGAGACCCGACTAGTAGGGTCTCTGTTT 3′), with two *Bsa*I restriction enzyme sites and a *Spe*I restriction enzyme recognition site, into the *Bbs*I gRNA cloning site of pX330 resulting in pX330-gRNA-mod. The modified gRNA expression cassette with the U6 promoter and a U6 terminator was then amplified by PCR from pX330-gRNA-mod using primers gRNA_NheI_fwd and gRNA_NheI_rev generating restriction sites for *Nhe*I. The amplified PCR product was digested and inserted into the *Avr*II restriction sites downstream of the respective Cas9 expression cassettes of pShV-TRE-Cas9-TetOn3G and pShV-CBh-Cas9, resulting in the plasmids pShV-Tre-Cas9-TetOn3G-gRNA and pShV-CBh-Cas9-gRNA respectively. Schematic plasmid maps are pictured in Fig. 1A. Note that all oligonucleotides used in this study for generation of vectors are listed in Supplementary Table 2.

### Construction of the intermediate HCAdV-BAC for recombineering of CRISPR/Cas9 expression units into the HCAdV genome

The intermediate HCAdV-BAC pBHCA-eGFP-NSH-ccdb-amp was constructed by recombining a ccdB-amp expression cassette flanked by the homology arms of the intermediate shuttle plasmid into the original BAC pBHCA-eGFP. For this the ccdb-amp expression cassette was inserted into the pShV and subsequently the ccdb^27^-amp expression cassette flanked by the homology arms was amplified by PCR using primers HR_sh_fwd and HR_SH_rev (Supplementary Table 2) generating 50 bp overhangs that served as homology arms for site-specific homologous recombination into pBHCA-eGFP. Briefly recombination was achieved by electroporating the purified PCR product into *E. coli* GBO5 that contained the pBHCA-eGFP and pSC101 plasmid carrying an arabinose inducible RecA. Upon arabinose induction the ccdb-amp expression cassette flanked by homology arms was recombined into the pBHCA-eGFP target site resulting in pHCAdV-eGFP-HOM-CCDB-AMP-HOM (Fig. 1C)^27^. Clones were selected on LB plates containing Ampicillin (Amp) and Chloramphenicol (Chl). Single clones were analyzed by restriction enzyme digest of isolated BACs. Bacteria of one positive clone were stored at −80 °C as glycerol stock until further recombineering steps were performed.

### Applying the intermediate shuttle plasmids and pHCA-eGFP-HOM-CCBD-AMP-HOM or pAdFTC for the generation of CRISPR/Cas9 HCAdV genomes

The HPV 18 *E6* specific gRNA t1 sequence (Supplementary Table 1) was previously published^29^. Forward and reverse DNA oligonucleotides with respective overhangs compatible to the oligonucleotide insertion site within pShV-CBh-Cas9-gRNA were synthesized (Eurofins). The double stranded gRNA oligonucleotide was introduced into the plasmid pShV-CBh-Cas9-gRNA following a previously published protocol^47^ as depicted in Fig. 1B with modifications described as follows. Briefly single-stranded DNA oligonucleotides were phosphorylated and annealed in a total volume of 10 µl using 100 µM of the forward and reverse oligonucleotides, 1ul 10x T4 ligase buffer (New England Biooabs) and 0.5 µl polynucleotide kinase (New England Biolabs). The reaction was incubated at 37 °C for 30 min followed by heating at 95 °C for 5 min and finally cooled down to 10 °C using a cooling rate of 0.1 °C/s. Phosphorylated double-stranded oligonucleotides were diluted 1:250 and 2 µl were mixed with 100ng pShV-CBh-Cas9-gRNA, 2ul 10x Buffer 2.1 (New England Biolabs), 1 mM ATP, 1ul *Bsa*I (New England Biolabs) and 0.5ul T4 DNA ligase (New England Biolabs) in a total volume of 20 µl. The reaction was cycled 6 times in a thermocycler at 37 °C for 5 min and 23 °C for 5 minutes. Ligation products were transformed into *E. coli* DH10α and selected on kanamycin containing LB-plates. Correct clones were confirmed by restriction enzyme digest and sequencing. The resulting plasmid was designated pShV-CBh-Cas9-gRNAHPV18 *E6*.

A double gRNA shuttle plasmid was constructed using the oligonucleotides Cr1 and Cr5 (Supplementary Table 1) specific for intronic sequences flanking exon 51 of the human dystrophin gene^30^. Cr1 and Cr5 were introduced into pShV-CBh-Cas9-gRNA, as described above, resulting in pShV-CBh-Cas9-gRNACr1 and pShV-CBh-Cas9-gRNACr5. The gRNACr1 expression unit was amplified by PCR from pShV-CBh-Cas9-gRNACr1 using primers gRNA_NheI_fwd and gRNA_NheI_rev. According to Fig. 1B the amplified PCR product was then ligated into the *Nhe*I restriction enzyme site within pShV-CBh-Cas9-gRNACr5 resulting in the plasmid pShV-CBh-Cas9-gRNACr5-Cr1.

The CCR5 specific gRNA oligonucleotide was predicted using a CRISPR design tool (http://crispr.mit.edu/). The oligonucleotide designated CCR5-88 (Supplementary Table 1) was inserted into BbsI restriction site of pX330 and gRNACCR5-88 was amplified by PCR using the primers gRNA_NheI_fwd and gRNA_NheI_rev and subsequently inserted into pShV-Tre-Cas9-Teton3G and pShV-CBH-Cas9 as described above resulting in plasmids pShV-Tre-Cas9-Teton3G-gRNACCR5-88 and pShV-CBH-Cas9-gRNACCR5-88 respectively. Note that all oligonucleotides used in this study are listed in Supplementary Table 2.

The single gRNA shuttle plasmid pShV-CBh-Cas9-gRNAHPV18E6 was digested with I-*Ceu*I and PI-*Sce*I (New England Biolabs) to release the CRISPR/Cas9 expression cassette HOM-Cas9-gRNAHPV18E6-HOM. Meanwhile RecA expression from the pSC101 plasmid in *E. coli* GBO5 containing the pHCAdV-eGFP-HOM-CCDB-AMP-HOM was induced by addition of arabinose and bacteria were grown for 40 min before they were rendered electrocompetent by consecutive washing with ice cold ddH_2_O for three times^27^. Gel-purified DNA fragment HOM-Cas9-gRNAHPV18E6-HOM was recombined into the HCAdV genome within pHCA-eGFP-HOM-CCDB-AMP-HOM according to the scheme in Fig. 1C by electroporation into the arabinose induced electrocompetent bacteria^27^. Clones were selected on LB-plates containing chloramphenicol and analyzed by a gRNA specific PCR using the primers gRNA_NheI_fwd and gRNA_NheI_rev as well as a restriction enzyme digest using I-*Ceu*I and PI-*Sce*I. Successful recombination resulted in respective HCADV-BAC designated pHCAdV-eGFP-CBh-Cas9-gRNAHPV18E6.

CRISPR/Cas9 expression cassettes from pShV-Tre-Cas9-gRNACCR5-88, pShV-CBH-Cas9-gRNACCR5-88 and pShV-CBH-Cas9-gRNACr5-Cr1 were cloned into I-*Ceu*I and PI-*Sce*I site of the HCAdV genome contained in pAdV-FTC^20^ as described before^31,33^. Slight modifications to accelerate the protocol are briefly described as follows. The shuttle plasmids containing respective gRNAs and pAdFTC were each co-digested with I-*Ceu*I and PI-*Sce*I to release the Cas9 and gRNA expression cassette/s from the shuttle plasmid. Linearized pAdFTC where purified using phenol-chloroform (Ph-Cl) extraction followed by ethanol (EtOH) precipitation. The shuttle plasmid backbone and the CBh-Cas9-gRNA expression cassette were separated on an agarose gel and the band containing the Cas9 and gRNA expression cassette(s) were gel-purified using the Wizard Gel- and PCR- clean-up kit (Promega). I-*Ceu*I and PI-*Sce*I-digested pAdFTC was dephosphorylated by adding 1 µl calf intestinal alkaline phosphatase (New England Biolabs) and incubated for 1 h at 37 °C and subsequently purified by Ph-Cl extraction followed by EtOH precipitation. Finally CBh-Cas9-gRNA or the Tre-Cas9-TetOn3G-gRNA expression cassette were ligated into linearized and dephosphorylated pAdFTC using 400U of T4 DNA ligase (New England Biolabs) and at a molar ratio of vector to insert of 1:3, respectively. Ligation reaction was incubated at 16 °C overnight and 4 µl of the ligation reaction were electroporated into DH10α electrocompetent *E. coli.* Bacteria were selected on ampicillin-containing LB plates. Clones were analyzed by restriction digest. Correct clones were selected and designated pAdFTC-Tre-Cas9-Teton3G-gRNACCR5-88, pAdFTC-CBH-Cas9-gRNACCR5-88, and pAdFTC-CBh-Cas9-gRNACr5-Cr1, respectively.

### Vector production and purification

HCAdV were produced following a previously published protocol^31,33^. The vector production procedure is illustrated in Supplementary Figure 4. Note that only changes to the previously published protocols are described here. The HCAdV genome contained in pBHCA-eGFP-CBh-Cas9-gRNAHPV18E6 was released from the BAC backbone by co-digestion using *Afe*I and *Hpa*I. HCAdV contained in pAdFTC-Tre-Cas9-Teton3G-gRNACCR5-88, pAdFTC-CBH-Cas9-gRNACCR5-88, and pAdFTC-CBh-Cas9-gRNACr5-Cr1 were released from the plasmid backbone by *Not*I restriction digest. Released HCAdV genomes were purified by Ph-Cl extraction followed by EtOH precipitation. Released purified vector genomes were transfected into the 116 producer cells and cells were co- transduced with helper virus (HV) AdNG163R-2^32^ at MOI 5.

Vectors were amplified by serial passaging from a 6 cm tissue culture dish to a 6 cm dish, to a 10 cm dish, to a 15 cm dish to 4 × 15 cm dishes and finally to 15 × 15 cm dishes, with co-transduction with HV at MOI 2. Before transduction of the next passage, cells from 500 µl of each lysate were collected for q-PCR based monitoring of HCAdV- amplification rates. Lysates from 15 × 15 cm dishes where used for vector purification using cesium chloride (CsCl) density gradient ultracentrifugation. Resulting HCAdV bands where harvested from the gradients and buffer was exchanged using PD-10 columns (GE Healthcare,) according to manufacturer’s instructions using an equilibration buffer (10 mM Tris-HCl (pH 7.5), 1 mM MgCl_2_) and elution buffer (10 mM Tris-HCl (pH 7.5), 1 mM MgCl_2,_ 10% glycerol) serving as buffer for long-term storage at −80 °C. Quantitative PCR based monitoring of HCAdV amplification and determination of the number of transducing vector units and physical particle numbers in final vector preparations was performed as previously described^31,33^.

### Cells and cell culture conditions

Hela and A549 cells were grown in DMEM medium (PAN Biotech, Aidenbach, Germany) supplemented with 10% fetal bovine serum (FBS, GE Healthcare) and 100 µg/ml Penicillin/Streptomycin (Pen/Strep, PAN Biotech). 116 cells were grown in MEM eagle medium (PAN Biotech) supplemented with 10% FBS, 100 µg/ml penicillin/streptomycin and 100 mg/ml hygromycin B (PAN Biotech). Primary human skeletal myoblasts (HSKM) were grown in DMEM supplemented with 20% FBS, 1% gentamycin and 1x Glutamax (Thermo Fischer Scientific). All cells were cultured at 37 °C and 5% CO_2_ in a standard tissue culture incubator.

### Viral delivery and detection of CRISPR/Cas9 mediated DNA double-strand break induction

Cells grown to confluency in 24 well plates were transduced with CRISPR-HCAdV at different multiplicities of infection (MOI). Hela cells were transduced with the vector HCAdV-eGFP-CBh-Cas9-gRNAHPV18E6 and A549 supplemented with 100ng/ml doxycycline were infected with the vector HCAdV-Tre-Cas9-Teton3G-gRNACCR5-88. For HCAdV-CBH-Cas9-gRNACr5-Cr1 primary human skeletal myoblasts were transduced.

48h–96h post transduction, cells were harvested and gDNA was extracted using the peqGold tissue DNA Mini Kit (Peqlab-VWR). DNA loci surrounding respective gRNA target sites were amplified by PCR. To detect mutations that were induced at the gRNAHPV18E6 binding site, primers HPV18T7E1_fwd and HPV18E6_rev were applied. For detection of mutations that were induced at the gRNA binding site of gRNACCR5-88 we used primers CCR5-fwd and CCR5_rev3. PCR products of the loci surrounding the HPV18E6 and CCR5-specific gRNA binding sites were subjected to T7E1 (New England Biolabs) mutation detection assay according to manufacturer’s instructions^48^. The dystrophin exon 51 deletion in cells treated with HCAdV-CBH-Cas9-gRNACr5-Cr1 was detected using primers CelI-CR1-F and CelI-CR5-R^30^. All primer sequences for mutation detection are listed in Supplementary Table 2. CRISPR/Cas9 efficiencies were quantified by measuring band intensity on agarose gels.

To show activity of the novel gRNA gRNA-CCR5-8 500 ng of the plasmid pX330-CCR5-88 were transfected into A549 cells and 3 days post-transfection genomic DNA (gDNA) was isolated. Subsequently the T7E1 mutation detection assay was performed as described above.

## Electronic supplementary material

Supplementary information
