# Supplementary material for: CRISPR/Cas9 delivery with one single adenoviral vector devoid of all viral genes
Source: Sci Rep. 2017 Dec 7;7:17113. doi: 10.1038/s41598-017-17180-w (PMC5719366; doi:10.1038/s41598-017-17180-w)
Supplement: Supplementary file 1 — Supplementary information [file 41598_2017_17180_MOESM1_ESM.pdf]

Supplemental information

CRISPR/Cas9 delivery with one single adenoviral vector devoid of all viral genes

Eric Ehrke- Schulz[1], Maren Schiwon[1], Theo Leitner[1], Stephan Dávid[1], Thorsten Bergmann[1], Jing Liu[1], Anja Ehrhardt[1]

[1]Institute of Virology and Microbiology, Center for Biomedical Education and Research (ZBAF), Department of Human Medicine, Faculty of Health, University of Witten/Herdecke, Witten, Germany

*Correspondence should be addressed to:

Anja Ehrhardt, Phone: +492302926273, Fax: +492302926 44278, Email: anja.ehrhardt@uni-wh.de

Chair of Virology and Microbiology

Center for Biomedical Education and Research (ZBAF)

Department of Human Medicine, Faculty of Health

Witten/Herdecke University

58453, Witten

Germany

Supplementary Table 1: Sequences of gRNA oligonucleotides used in this study

Oligo name		Sequence	
**t1 (1)**	5’	gGCGCTTTGAGGATCCAACA	3’
**CCR5-88**	5’	gTCACTATGCTGCCGCCCAGT	3’
**Cr1 (2)**	5’	CATTGGCTTTGATTTCCCTA	3’
**Cr5 (2)**	5’	CCAGTTGCCTAAGAACTGGT	3’

Supplementary Table 2: Primers used in this study

Primer name		Sequence	
**Cas9_ fwd_AccI**	5’	ATGCAACTGCAGTAGTCGAC	3’
**Cas9_rev_XmaI**	5’	ATCCCGGGCTCCCCAGCATGCCTGC	3’
**Tet3G_PacI_fwd**	5’	ATTAATTAACAGAGTAATTCATACAAAAGGACTCGC	3’
**Tet3G_PacI_rev**	5’	ATTAATTAATTGGTCGAGCTGGATACTTCC3’	3’
**gRNA NheI fwd**	5’	ATGCTAGCGAGGGCCTATTTCCCATGATTCC	3’
**gRNA NheI rev**	5’	ATGCTAGCCTGCAGAATTGGCGCACG	3’
**HR sh fwd**	5’	GGTGGACTCACAGGCCATTCTGCTTTTATTTGGTCAAC CTCAGTTCACAATAACTATAACGGTCCTAAGGTAG	3’
**HR SH rev**	5’	GAACTCTCCTTGAAGTGGGCAGATTACCCTTTGAATAA CGTTCTATCCCGTGCCATTTCATTACCTCTTTCTC	3’
**gRNA BamHI fwd**	5’	ACTGGGATCCGAGGGCCTATTTCCCATGATTCC	3’
**gRNA BamHI rev**	5’	ACGTGGATCCCTGCAGAATTGGCGCACG	3’
**HPV18T7E1fwd**	5’	CTTGCATAACTATATCCACTCCC	3’
**HPV18E6_rev**	5’	ATTCAACGGTTTCTGGCAC	3’
**CCR5_fwd**	5’	AGATGGATTATCAAGTGTCAAGTCC	3’
**CCR5_rev3**	5’	GATGACCATGACAAGCAGC	3’
**Cell-CR1-F (2)**	5’	GAGAGGTTATGTGGCTTTACCA	3’
**Cell-CR5-R-(2)**	5’	CTGCGTAGTGCCAAAACAAA	3’

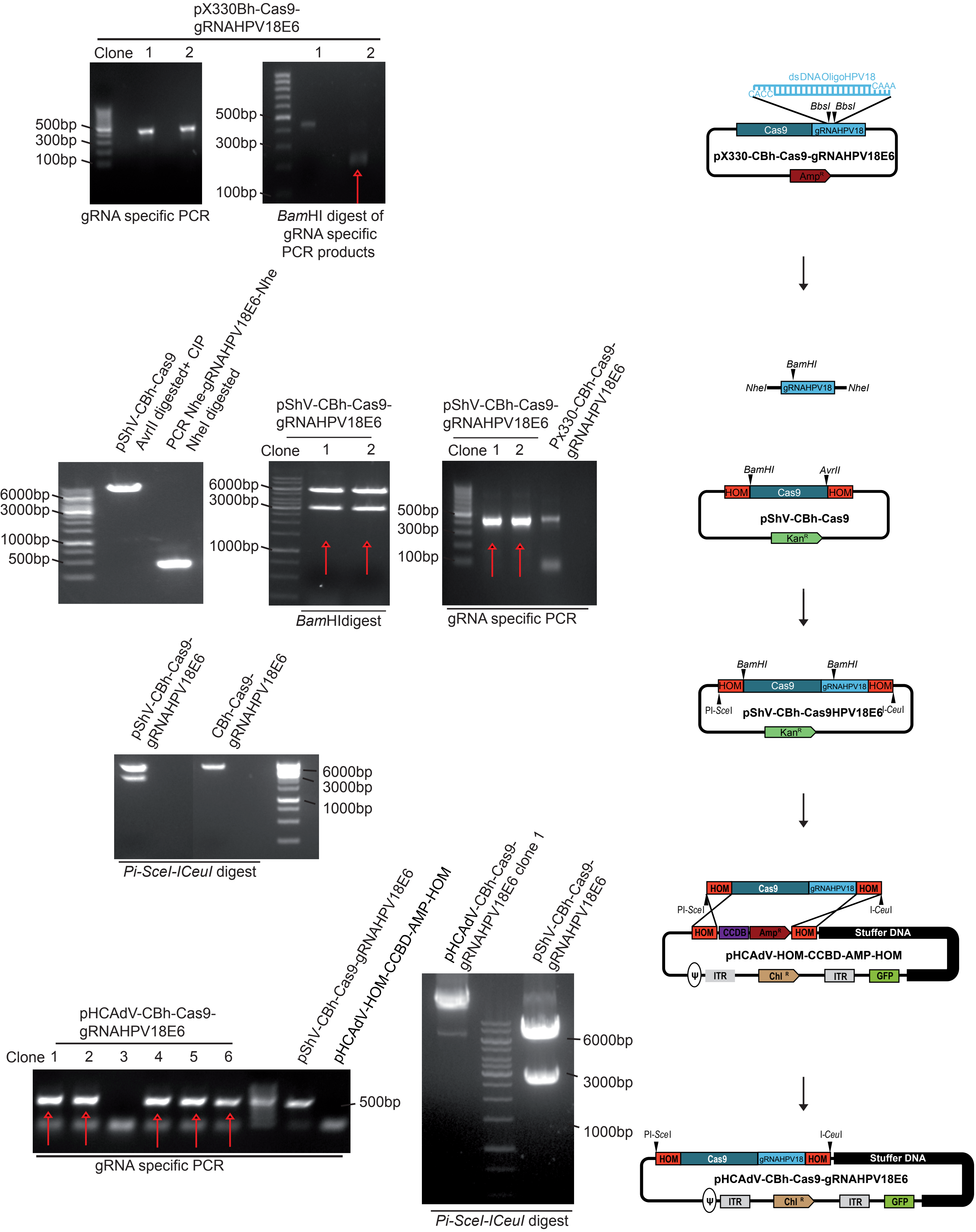

Supplementary Figure 1: Cloning of HCAdV-CBh-Cas9-gRNAHPV18E6. The cloning of the construct was performed as schematically shown on the right panel. Representative agarose gel pictures of each cloning step are depicted on the left. Positive clones are indicated by red arrows.

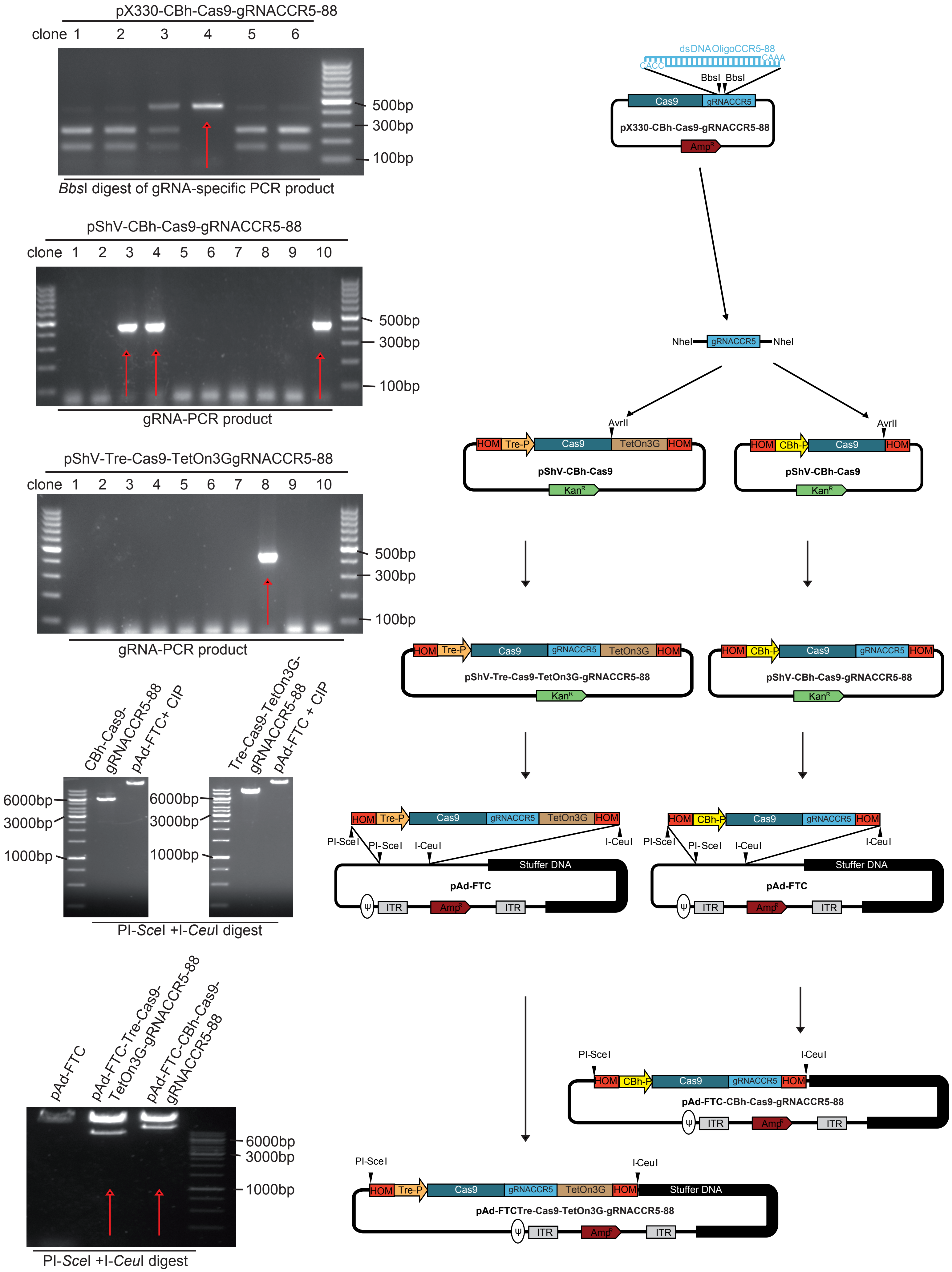

Supplementary Figure 2: Cloning of HCAdV-CBh-Cas9-gRNACCR5-88 and HCAdV-TRE-Cas9-TetOn3G-gRNACCR5-88: The cloning of these constructs was done as schematically shown on the right panel. Representative agarose gel pictures of each cloning step are depicted on the left. Positive clones are indicated by red arrows.

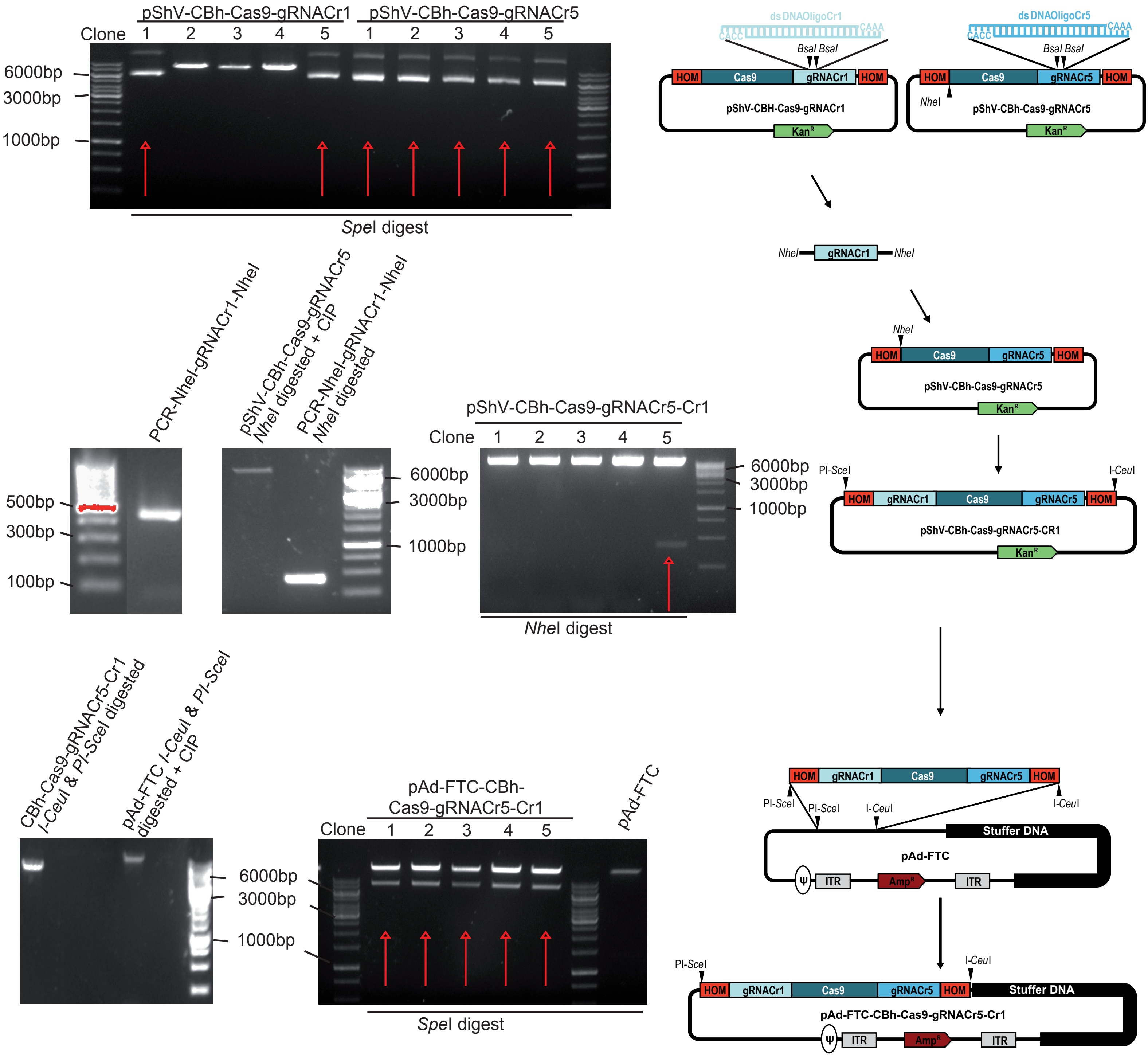

Supplementary Figure 3: Cloning of HCAdV-CBh-Cas9-gRNACr5-Cr1. The cloning of the construct was done as schematically shown on the right panel. Representative agarose gel pictures of each cloning step are depicted on the left. Positive clones are indicated by red arrows.

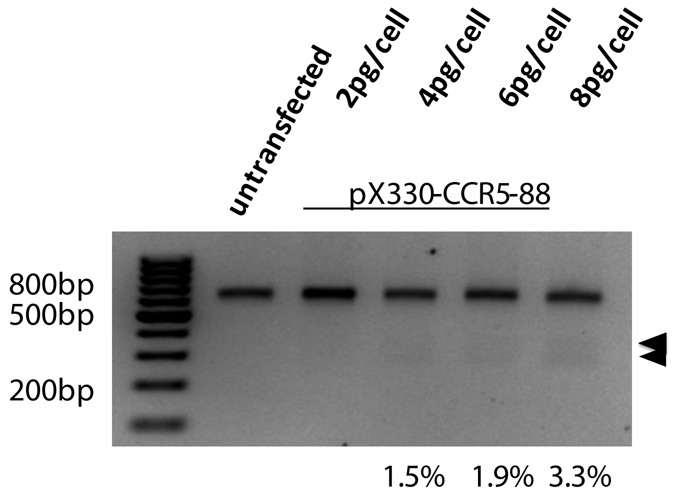

Supplementary Figure 4: **Functionality tests after plasmid transfection of pX330-CCR5-88 in A549 cells.** Cells were transfected with increasing amounts of plasmid DNA(2-8pg/cell). Two days after transfection genomic DNA was extracted and subjected to T7E1 assay**.** For functional gRNAs cleavage products of 278bp and 360bp were expected. Mutat onrates of CRISPR Cas9 at the CCR5 locus are shown below the image.

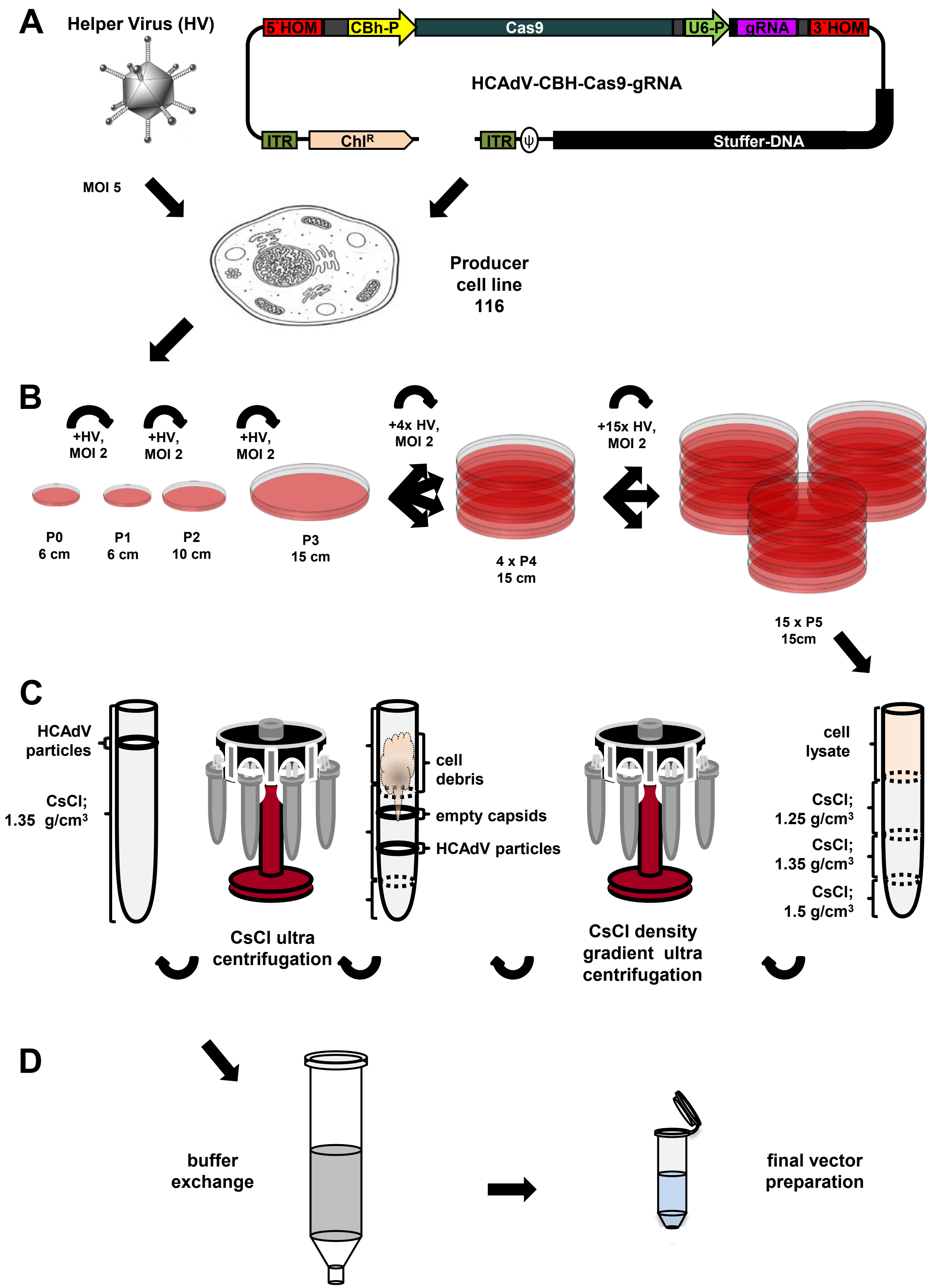

Supplementary Figure 5: Accelerated medium scale HCAdV amplification and purification. (A) Transfection of linearized HCAdV genome containing customized CRSIPR/Cas9 expression units into the HCAdV producer cell line (116 cells) and subsequent infection with the HV AdNG163R-2. (B) Amplification of HCAdV by serial serial transfer of cell virus lysate to a new tissue culture dish and co-infecting with HV, lysate from 16x 15cm dishes was used for HCAdV purification. (C) HCAdV particles were purified by ultracentrifugation using CsCl gradients. (D) Buffer exchange of purified HCAdV particles. HCAdV, high-capacity adenoviral vector; ITR, adenovirus serotype 5 inverted terminal repeat; Ψ, packaging signal; HV, helper virus; MOI, multiplicity of infection; P, passage, CsCl, caesium chloride.

Supplementary information: Complete sequence of the Shuttle plasmid pShV-TRE-Cas9-TetOn3G-gRNA

LOCUS Exported 10895 bp ds-DNA circular SYN 23-JUL-2015
DEFINITION .
ACCESSION .
VERSION .
KEYWORDS Finished Plasmid Cloned pShV Cas9 TRE gRNA Bsa1 Spe1 scaffold
TET... SOURCE synthetic DNA construct
ORGANISM recombinant plasmid
REFERENCE 1 (bases 1 to 10895)
AUTHORS Theo
TITLE Direct Submission
JOURNAL Exported Donnerstag, 9. Jun 2016 from SnapGene 2.5.0
http://www.snapgene.com
FEATURES Location/Qualifiers
source 1..10895
/organism="recombinant plasmid"
/mol_type="other DNA"
CDS complement(385..1179)
/codon_start=1
/gene="aph(3')-II (or nptII)"
/product="aminoglycoside phosphotransferase from Tn5"
/note="NeoR/KanR"
/note="confers resistance to neomycin, kanamycin, and G418
(Geneticin(R))"
/translation="MIEQDGLHAGSPAAWVERLFGYDWAQQTIGCSDAAVFRLSAQGRP
VLFVKTDLSGALNELQDEAARLSWLATTGVPCAAVLDVVTEAGRDWLLLGEVPGQDLLS
SHLAPAEKVSIMADAMRRLHTLDPATCPFDHQAKHRIERARTRMEAGLVDQDDLDEEHQ
GLAPAELFARLKARMPDGEDLVVTHGDACLPNIMVENGRFSGFIDCGRLGVADRYQDIA
LATRDIAEELGGEWADRFLVLYGIAAPDSQRIAFYRLLDEFF"
rep_origin 1481..2069
/direction=RIGHT
/note="ori"
/note="high-copy-number ColE1/pMB1/pBR322/pUC origin of
replication"
misc_feature 2468..2611
/note="sequence confirmed"
misc_feature 2468..2494
/note="Pi-SceI"
misc_feature 2495..2610
/note="left homology arm"
misc_feature 2611
/note="MCS"
promoter 2617..2861
/note="Human U6 Promoter"
misc_feature 2890..2965
/note="chimeric guide RNA scaffold"
misc_feature 2966..2971
/note="U6 terminator"
misc_feature 3042..3092
/note="sequence confirmed"
misc_feature 3042..3091
/note="MCS"
misc_feature 3095..3326
/note="bGH polyA"
misc_feature 3336..3383
/note="NLS"

misc_feature 3384..7484
/note="Cas9"
/note="hSpCsn1"
misc_feature 7485..7535
/note="NLS"
misc_feature 7536..7604
/note="3xFLAG-Tag"
/note="3xFLAG"
misc_feature 7616..8006
/note="sequence confirmed"
misc_feature complement(7619..7994)
/label="TRE3G Promoter"
/note="TRE3G Promoter"
misc_feature 7997..8006
/note="new MCS"
misc_feature 8012..9346
/note="Human EF1a Promoter"
misc_feature 9454..10200
/note="Tet-On 3G"
polyA_signal 10223..10665
/note="SV40 polyA signal"
misc_feature 10684..10686
/note="sequence confirmed"
misc_feature 10687..10895
/note="sequence confirmed 2"
misc_feature 10687..10747
/note="2nd MCS"
misc_feature 10748..10859
/note="right homology arm"
misc_feature 10860..10895
/note="I-CeuI"
ORIGIN
1 catttaattg gtgcactctc agtacaatct gctctgatgc cgcatagtta agccagcccc
61 gacacccgcc aacacccgct gacgcgccct gacgggcttg tctgctcccg gcatccgctt
121 acagacaagc tgtgaccgtc tccgggagct gcatgtgtca gaggttttca ccgtcatcac
181 cgaaacgcgc gagacgaaag ggcctcgtga tacgcctatt tttataggtt aatgtcatga
241 taataatggt ttcttagacg tcaggtggca cttttcgggg aaatgtgcgc ggaaccccta
301 tttgtttatt tttctaaata cattcaaata tgtatccgct catgagacaa taaccctgat
361 aaatgcttca ataatattac cggttcagaa gaactcgtca agaaggcgat agaaggcgat
421 gcgctgcgaa tcgggagcgg cgataccgta aagcacgagg aagcggtcag cccattcgcc
481 gccaagctct tcagcaatat cacgggtagc caacgctatg tcctgatagc ggtccgccac
541 acccagccgg ccacagtcga tgaatccaga aaagcggcca ttttccacca tgatattcgg
601 caagcaggca tcgccatggg tcacgacgag atcctcgccg tcgggcatgc gcgccttgag
661 cctggcgaac agttcggctg gcgcgagccc ctgatgctct tcgtccagat catcctgatc
721 gacaagaccg gcttccatcc gagtacgtgc tcgctcgatg cgatgtttcg cttggtggtc
781 gaatgggcag gtagccggat caagcgtatg cagccgccgc attgcatcag ccatgatgga
841 tactttctcg gcaggagcaa ggtgggatga caggagatcc tgccccggca cttcgcccaa
901 tagcagccag tcccttcccg cttcagtgac aacgtcgagc acagctgcgc aaggaacgcc
961 cgtcgtggcc agccacgata gccgcgctgc ctcgtcctgc agttcattca gggcaccgga
1021 caggtcggtc ttgacaaaaa gaaccgggcg cccctgcgct gacagccgga acacggcggc
1081 atcagagcag ccgattgtct gttgtgccca gtcatagccg aatagcctct ccacccaagc
1141 ggccggagaa cctgcgtgca atccatcttg ttcaatcatg cgaaacgatc ctcatcctgt
1201 ctcttgatca gatcttgatc ccctgcgcca tcagatcctt ggcggcaaga aagccatcca
1261 gtttactttg cagggcttcc caaccttacc agagggcgcc ccagctggca attccggaat
1321 atttttactc atatatactt tagattgatt taaaacttca tttttaattt aaaaggatct
1381 aggtgaagat cctttttgat aatctcatga ccaaaatccc ttaacgtgag ttttcgttcc
1441 actgagcgtc agaccccgta gaaaagatca aaggatcttc ttgagatcct ttttttctgc

1501 gcgtaatctg ctgcttgcaa acaaaaaaac caccgctacc agcggtggtt tgtttgccgg
1561 atcaagagct accaactctt tttccgaagg taactggctt cagcagagcg cagataccaa
1621 atactgtcct tctagtgtag ccgtagttag gccaccactt caagaactct gtagcaccgc
1681 ctacatacct cgctctgcta atcctgttac cagtggctgc tgccagtggc gataagtcgt
1741 gtcttaccgg gttggactca agacgatagt taccggataa ggcgcagcgg tcgggctgaa
1801 cggggggttc gtgcacacag cccagcttgg agcgaacgac ctacaccgaa ctgagatacc
1861 tacagcgtga gctatgagaa agcgccacgc ttcccgaagg gagaaaggcg gacaggtatc
1921 cggtaagcgg cagggtcgga acaggagagc gcacgaggga gcttccaggg ggaaacgcct
1981 ggtatcttta tagtcctgtc gggtttcgcc acctctgact tgagcgtcga tttttgtgat
2041 gctcgtcagg ggggcggagc ctatggaaaa acgccagcaa cgcggccttt ttacggttcc
2101 tggccttttg ctggcctttt gctcacatgt tctttcctgc gttatcccct gattctgtgg
2161 ataaccgtat taccgccttt gagtgagctg ataccgctcg ccgcagccga acgaccgagc
2221 gcagcgagtc agtgagcgag gaagcggaag agcgcccaat acgcaaaccg cctctccccg
2281 cgcgttggcc gattcattaa tgcagctggc acgacaggtt tcccgactgg aaagcgggca
2341 gtgagcgcaa cgcaattaat gtgagttagc tcactcatta ggcaccccag gctttacact
2401 ttatgcttcc ggctcgtatg ttgtgtggaa ttgtgagcgg ataacaattt cacacaggaa
2461 ttaaatgtaa ctataacggt cctaaggtag cgaaagatct atgacatcgt tatactctta
2521 ctgcatcagc actgtcctgc tccttgtgat gttacaaaga ccggctttca accactcacc
2581 atctctccga aagacattat tattcctcca cctagcgagg gcctatttcc catgattcct
2641 tcatatttgc atatacgata caaggctgtt agagagataa ttggaattaa tttgactgta
2701 aacacaaaga tattagtaca aaatacgtga cgtagaaagt aataatttct tgggtagttt
2761 gcagttttaa aattatgttt taaaatggac tatcatatgc ttaccgtaac ttgaaagtat
2821 ttcgatttct tggctttata tatcttgtgg aaaggacgaa acaccggaga cccgactagt
2881 agggtctctg ttttagagct agaaatagca agttaaaata aggctagtcc gttatcaact
2941 tgaaaaagtg gcaccgagtc ggtgcttttt tgttttagag ctagaaatag caagttaaaa
3001 taaggctagt ccgtttttag cgcgtgcgcc aattctgcag gctaggcacg tagtgagtac
3061 tcgcgacccg ggggcatgca actgcagtag tcgctcccca gcatgcctgc tattctcttc
3121 ccaatcctcc cccttgctgt cctgccccac cccacccccc agaatagaat gacacctact
3181 cagacaatgc gatgcaattt cctcatttta ttaggaaagg acagtgggag tggcaccttc
3241 cagggtcaag gaaggcacgg gggaggggca aacaacagat ggctggcaac tagaaggcac
3301 agtcgaggct gatcagcgag ctctaggaat tcttactttt tcttttttgc ctggccggcc
3361 tttttcgtgg ccgccggcct tttgtcgcct cccagctgag acaggtcgat ccgtgtctcg
3421 tacaggccgg tgatgctctg gtggatcagg gtggcgtcca gcacctcttt ggtgctggtg
3481 tacctcttcc ggtcgatggt ggtgtcaaag tacttgaagg cggcaggggc tcccagattg
3541 gtcagggtaa acaggtggat gatattctcg gcctgctctc tgatgggctt atcccggtgc
3601 ttgttgtagg cggacagcac tttgtccaga ttagcgtcgg ccaggatcac tctcttggag
3661 aactcgctga tctgctcgat gatctcgtcc aggtagtgct tgtgctgttc cacaaacagc
3721 tgtttctgct cattatcctc gggggagccc ttcagcttct catagtggct ggccaggtac
3781 aggaagttca catatttgga gggcagggcc agttcgtttc ccttctgcag ttcgccggca
3841 gaggccagca ttctcttccg gccgttttcc agctcgaaca gggagtactt aggcagcttg
3901 atgatcaggt ccttttttcac ttctttgtag cccttggctt ccagaaagtc gatgggattc
3961 ttctcgaagc tgcttctttc catgatggtg atccccagca gctctttcac actcttcagt
4021 ttcttggact tgcccttttc cactttggcc accaccagca cagaataggc cacggtgggg
4081 ctgtcgaagc cgccgtactt cttagggtcc cagtccttct ttctggcgat cagcttatcg
4141 ctgttcctct tgggcaggat agactctttg ctgaagccgc ctgtctgcac ctcggtcttt
4201 ttcacgatat tcacttgggg catgctcagc actttccgca cggtggcaaa atcccggccc
4261 ttatcccaca cgatctcccc ggtttcgccg tttgtctcga tcagaggccg cttccggatc
4321 tcgccgttgg ccagggtaat ctcggtcttg aaaaagttca tgatgttgct gtagaagaag
4381 tacttggcgg tagccttgcc gatttcctgc tcgctcttgg cgatcatctt ccgcacgtcg
4441 tacaccttgt agtcgccgta cacgaactcg ctttccagct tagggtactt tttgatcagg
4501 gcggttccca cgacggcgtt caggtaggcg tcgtgggcgt ggtggtagtt gttgatctcg
4561 cgcactttgt aaaactggaa atccttccgg aaatcggaca ccagcttgga cttcagggtg
4621 atcactttca cttcccggat cagcttgtca ttctcgtcgt acttagtgtt catccgggag
4681 tccaggatct gtgccacgtg ctttgtgatc tgccgggttt ccaccagctg tctcttgatg
4741 aagccggcct tatccagttc gctcaggccg cctctctcgg ccttggtcag attgtcgaac
4801 tttctctggg taatcagctt ggcgttcagc agctgccgcc agtagttctt catcttcttc
4861 acgacctctt cggagggcac gttgtcgctc ttgccccggt tcttgtcgct tctggtcagc

4921 accttgttgt cgatggagtc gtccttcaga aagctctgag gcacgatatg gtccacatcg
4981 tagtcggaca gccggttgat gtccagttcc tggtccacgt acatatcccg cccattctgc
5041 aggtagtaca ggtacagctt ctcgttctgc agctgggtgt tttccacggg gtgttctttc
5101 aggatctggc tgcccagctc tttgatgccc tcttcgatcc gcttcattct ctcgcggctg
5161 ttcttctgtc ccttctgggt ggtctggttc tctctggcca tttcgatcac gatgttctcg
5221 ggcttgtgcc ggcccatcac tttcacgagc tcgtccacca ccttcactgt ctgcaggatg
5281 cccttcttaa tggcggggct gccggccaga ttggcaatgt gctcgtgcag gctatcgccc
5341 tggccggaca cctgggcttt ctggatgtcc tctttaaagg tcaggctgtc gtcgtggatc
5401 agctgcatga agtttctgtt ggcgaagccg tcggacttca ggaaatccag gattgtcttg
5461 ccggactgct tgtcccggat gccgttgatc agcttccggc tcagcctgcc ccagccggtg
5521 tatctccgcc gcttcagctg cttcatcact ttgtcgtcga acaggtgggc ataggttttc
5581 agccgttcct cgatcatctc tctgtcctca aacagtgtca gggtcagcac gatatcttcc
5641 agaatgtcct cgttttcctc attgtccagg aagtccttgt ccttgataat tttcagcaga
5701 tcgtggtatg tgcccaggga ggcgttgaac cgatcttcca cgccggagat ttccacggag
5761 tcgaagcact cgattttctt gaagtagtcc tctttcagct gcttcacggt cactttccgg
5821 ttggtcttga acagcaggtc cacgatggcc tttttctgct cgccgctcag gaaggcgggc
5881 tttctcattc cctcggtcac gtatttcact ttggtcagct cgttatacac ggtgaagtac
5941 tcgtacagca ggctgtgctt gggcagcacc ttctcgttgg gcaggttctt atcgaagttg
6001 gtcatccgct cgatgaagct ctgggcggaa gcgcccttgt ccaccacttc ctcgaagttc
6061 caggggggtga tggtttcctc gctctttctg gtcatccagg cgaatctgct gtttcccctg
6121 gccagagggc ccacgtagta ggggatgcgg aaggtcagga tcttctcgat cttttcccgg
6181 ttgtccttca ggaatgggta aaaatcttcc tgccgccgca gaatggcgtg cagctctccc
6241 aggtggatct ggtgggggat gctgccgttg tcgaaggtcc gctgcttccg cagcaggtcc
6301 tctctgttca gcttcacgag cagttcctcg gtgccgtcca tcttttccag gatgggcttg
6361 atgaacttgt agaactcttc ctggctggct ccgccgtcaa tgtagccggc gtagccgttc
6421 ttgctctggt cgaagaaaat ctctttgtac ttctcaggca gctgctgccg cacgagagct
6481 ttcagcaggg tcaggtcctg gtggtgctcg tcgtatctct tgatcataga ggcgctcagg
6541 ggggccttgg tgatctcggt gttcactctc aggatgtcgc tcagcaggat ggcgtcggac
6601 aggttcttgg cggccagaaa caggtcggcg tactggtcgc cgatctgggc cagcaggttg
6661 tccaggtcgt cgtcgtaggt gtccttgctc agctgcagtt tggcatcctc ggccaggtcg
6721 aagttgctct tgaagttggg ggtcaggccc aggctcaggg caatcaggtt tccgaacagg
6781 ccattcttct tctcgccggg cagctgggcg atcagatttt ccagccgtct gctcttgctc
6841 agtctggcag acaggatggc cttggcgtcc acgccgctgg cgttgatggg gttttcctcg
6901 aacagctggt tgtaggtctg caccagctgg atgaacagct tgtccacgtc gctgttgtcg
6961 gggttcaggt cgccctcgat caggaagtgg ccccggaact tgatcatgtg ggccagggcc
7021 agatagatca gccgcaggtc ggccttgtcg gtgctgtcca ccagtttctt tctcaggtgg
7081 tagatggtgg ggtacttctc gtggtaggcc acctcgtcca cgatgttgcc gaagatgggg
7141 tgccgctcgt gcttcttatc ctcttccacc aggaaggact cttccagtct gtggaagaag
7201 ctgtcgtcca ccttggccat ctcgttgctg aagatctctt gcagatagca gatccggttc
7261 ttccgtctgg tgtatcttct tctggcggtt ctcttcagcc gggtggcctc ggctgtttcg
7321 ccgctgtcga acagcagggc tccgatcagg ttcttcttga tgctgtgccg gtcggtgttg
7381 cccagcacct tgaatttctt gctgggcacc ttgtactcgt cggtgatcac ggcccagccc
7441 acagagttgg tgccgatgtc caggccgatg ctgtacttct tgtcggctgc tgggactccg
7501 tggataccga ccttccgctt cttctttggg gccatcttat cgtcatcgtc tttgtaatca
7561 atatcatgat ccttgtagtc tccgtcgtgg tccttatagt ccatggtggc accgggactt
7621 tacgagggta ggaagtggta cggaaagttg gtataagaca aaagtgttgt ggaattgctc
7681 caggcgatct gacggttcac taaacgagct ctgcttttat aggcgcccac cgtacacgcc
7741 taaagcttat acgttctcta tcactgatag ggagtaaact ggatatacgt tctctatcac
7801 tgatagggag taaactgtag atacgttctc tatcactgat agggagtaaa ctggtcatac
7861 gttctctatc actgataggg agtaaactcc ttatacgttc tctatcactg atagggagta
7921 aagtctgcat acgttctcta tcactgatag ggagtaaact cttcatacgt tctctatcac
7981 tgatagggag taaactcgac cttaattaac agagtaattc atacaaaagg actcgcccct
8041 gccttgggga atcccaggga ccgtcgttaa actcccacta acgtagaacc cagagatcgc
8101 tgcgttcccg ccccctcacc cgcccgctct cgtcatcact gaggtggaga agagcatgcg
8161 tgaggctccg gtgcccgtca gtgggcagag cgcacatcgc ccacagtccc cgagaagttg
8221 gggggagggg tcggcaattg aaccggtgcc tagagaaggt ggcgcggggt aaactgggaa
8281 agtgatgtcg tgtactggct ccgccttttt cccgagggtg ggggagaacc gtatataagt

8341 gcagtagtcg ccgtgaacgt tctttttcgc aacgggtttg ccgccagaac acaggtaagt
8401 gccgtgtgtg gttcccgcgg gcctggcctc tttacgggtt atggcccttg cgtgccttga
8461 attacttcca cgcccctggc tgcagtacgt gattcttgat cccgagcttc gggttggaag
8521 tgggtgggag agttcgaggc cttgcgctta aggagcccct tcgcctcgtg cttgagttga
8581 ggcctggctt gggcgctggg gccgccgcgt gcgaatctgg tggcaccttc gcgcctgtct
8641 cgctgctttc gataagtctc tagccattta aaatttttga tgacctgctg cgacgctttt
8701 tttctggcaa gatagtcttg taaatgcggg ccaagatctg cacactggta tttcggtttt
8761 tggggccgcg ggcggcgacg gggcccgtgc gtcccagcgc acatgttcgg cgaggcgggg
8821 cctgcgagcg cggccaccga gaatcggacg ggggtagtct caagctggcc ggcctgctct
8881 ggtgcctggc ctcgcgccgc cgtgtatcgc cccgccctgg gcggcaaggc tggcccggtc
8941 ggcaccagtt gcgtgagcgg aaagatggcc gcttcccggc cctgctgcag ggagctcaaa
9001 atggaggacg cggcgctcgg gagagcgggc gggtgagtca cccacacaaa ggaaaagggc
9061 ctttccgtcc tcagccgtcg cttcatgtga ctccacggag taccgggcgc cgtccaggca
9121 cctcgattag ttctcgagct tttggagtac gtcgtcttta ggttgggggg aggggtttta
9181 tgcgatggag tttccccaca ctgagtgggt ggagactgaa gttaggccag cttggcactt
9241 gatgtaattc tccttggaat ttgccctttt tgagtttgga tcttggttca ttctcaagcc
9301 tcagacagtg gttcaaagtt tttttcttcc atttcaggtg tcgtgagagc tcgtttagtg
9361 aaccgtcaga tcgcctggag acgccatcca cgctgttttg acctccatag aagacaccgg
9421 gaccgatcca gcctccgcgg ccccgaattc accatgtcta gactggacaa gagcaaagtc
9481 ataaactctg ctctggaatt actcaatgga gtcggtatcg aaggcctgac gacaaggaaa
9541 ctcgctcaaa agctgggagt tgagcagcct accctgtact ggcacgtgaa gaacaagcgg
9601 gccctgctcg atgccctgcc aatcgagatg ctggacaggc atcataccca ctcctgcccc
9661 ctggaaggcg agtcatggca agactttctg cggaacaacg ccaagtcata ccgctgtgct
9721 ctcctctcac atcgcgacgg ggctaaagtg catctcggca cccgcccaac agagaaacag
9781 tacgaaaccc tggaaaatca gctcgcgttc ctgtgtcagc aaggcttctc cctggagaac
9841 gcactgtacg ctctgtccgc cgtgggccac tttacactgg gctgcgtatt ggaggaacag
9901 gagcatcaag tagcaaaaga ggaaagagag acacctacca ccgattctat gcccccactt
9961 ctgaaacaag caattgagct gttcgaccgg cagggagccg aacctgcctt ccttttcggc
10021 ctggaactaa tcatatgtgg cctggagaaa cagctaaagt gcgaaagcgg cgggccgacc
10081 gacgcccttg acgattttga cttagacatg ctcccagccg atgcccttga cgactttgac
10141 cttgatatgc tgcctgctga cgctcttgac gattttgacc ttgacatgct ccccgggtaa
10201 ctaagtaagg atccagacat gataagatac attgatgagt ttggacaaac cacaactaga
10261 atgcagtgaa aaaaatgctt tatttgtgaa atttgtgatg ctattgcttt atttgtaacc
10321 attataagct gcaataaaca agttaacaac aacaattgca ttcattttat gtttcaggtt
10381 cagggggagg tgtgggaggt tttttaaagc aagtaaaacc tctacaaatg tggtatggct
10441 gattatgatc ctgcaagcct cgtcgtcctg gccggaccac gctatctgtg caaggtcccc
10501 ggccccggac gcgcgctcca tgagcagagc gcccgccgcc gaggcgaaga ctcgggcggc
10561 gccctgcccg tcccaccagg tcaacaggcg gtaaccggcc tcttcatcgg gaatgcgcgc
10621 gaccttcagc atcgccggca tgtccccctg gcggacggga agtatccagc tcgaccaatt
10681 aattaagtct agatcggatc caaggtaccc tgatcgatat cgagctcttg ctagcagtac
10741 tgaattcgca ttcacattca atataagtgc cactatcagc aatctatcta tcccactaac
10801 tggatagaaa catcgttatt cagccatttg taagagaaga cgcaatagga acatcaagat
10861 ctatgtcggg tgcggagaaa gaggtaatga aatgg//

Supplementary informaiton: Complete sequence of the Shuttle plasmid pShV-CBh-Cas9-gRNA

```
LOCUS       Exported               8651 bp ds-DNA     circular SYN 26-OKT-2015
DEFINITION  .
ACCESSION   .
VERSION     .
KEYWORDS    pShV CbH Cas9
GoldenGate SOURCE      synthetic DNA
construct ORGANISM  recombinant plasmid
REFERENCE   1  (bases 1 to 8651)
  AUTHORS   Theo
  TITLE     Direct Submission
  JOURNAL   Exported Donnerstag, 9. Jun 2016 from SnapGene 2.5.0
            http://www.snapgene.com
FEATURES             Location/Qualifiers
     source          1..8651
                     /organism="recombinant plasmid"
                     /mol_type="other DNA"
     CDS             303..1097
                     /codon_start=1
                     /gene="aph(3')-II (or nptII)"
                     /product="aminoglycoside phosphotransferase from Tn5"
                     /note="NeoR/KanR"
                     /note="confers resistance to neomycin, kanamycin, and G418
                     (Geneticin(R))"
                     /translation="MIEQDGLHAGSPAAWVERLFGYDWAQQTIGCSDAAVFRLSAQGRP
                     VLFVKTDLSGALNELQDEAARLSWLATTGVPCAAVLDVVTEAGRDWLLLGEVPGQDLLS
                     SHLAPAEKVSIMADAMRRLHTLDPATCPFDHQAKHRIERARTRMEAGLVDQDDLDEEHQ
                     GLAPAELFARLKARMPDGEDLVVTHGDACLPNIMVENGRFSGFIDCGRLGVADRYQDIA
                     LATRDIAEELGGEWADRFLVLYGIAAPDSQRIAFYRLLDEFF"
     variation       335..340
                     /note="NaeI site?"
     misc_feature    1477..1480
                     /note="SwaI"
     misc_feature    1482..1517
                     /note="I-CeuI"
     misc_feature    1518..1629
                     /note="right homology arm"
     misc_feature    1630..1658
                     /note="MCS"
     misc_feature    1670..2468
                     /note="CBh"
     misc_feature    2481..2549
                     /note="3xFLAG"
     misc_feature    2550..2600
                     /note="NLS"
     misc_feature    2601..6701
                     /note="hSpCsn1"
     misc_feature    6702..6749
                     /note="NLS"
     misc_feature    6759..6990
                     /note="bGH polyA"
     misc_feature    6997..7087
                     /note="MCS"
     promoter        7093..7337
                     /note="Human U6 Promoter"
     misc_feature    7338..7369
```

```
                     /note="Bsa1 Cloning site"
     misc_feature    7370..7441
                     /note="chimeric guide RNA scaffold"
     misc_feature    7442..7447
                     /note="U6 terminator"
     misc_feature    7518..7522
                     /note="MCS"
     misc_feature    7523..7638
                     /note="left homology arm"
     misc_feature    7639..7665
                     /note="Pi-SceI"
     misc_feature    7667..7670
                     /note="SwaI"
     protein_bind    7686..7702
                     /bound_moiety="lac repressor encoded by lacI"
                     /note="lac operator"
                     /note="The lac repressor binds to the lac operator to
                     inhibit transcription in E. coli. This inhibition can be
                     relieved by adding lactose or
                     isopropyl-beta-D-thiogalactopyranoside (IPTG)."
     promoter        complement(7710..7740)
                     /note="lac promoter"
                     /note="promoter for the E. coli lac operon"
     protein_bind    7755..7776
                     /bound_moiety="E. coli catabolite activator protein"
                     /note="CAP binding site"
                     /note="CAP binding activates transcription in the presence
                     of cAMP."
     rep_origin      complement(8064..1)
                     /direction=LEFT
                     /note="ori"
                     /note="high-copy-number ColE1/pMB1/pBR322/pUC origin of
                     replication"
ORIGIN
        1 agaagatcct ttgatctttt ctacggggtc tgacgctcag tggaacgaaa actcacgtta
       61 agggattttg gtcatgagat tatcaaaaag gatcttcacc tagatccttt taaattaaaa
      121 atgaagtttt aaatcaatct aaagtatata tgagtaaaaa tattccggaa ttgccagctg
      181 gggcgccctc tggtaaggtt gggaagccct gcaaagtaaa ctggatggct ttcttgccgc
      241 caaggatctg atggcgcagg ggatcaagat ctgatcaaga gacaggatga ggatcgtttc
      301 gcatgattga acaagatgga ttgcacgcag gttctccggc cgcttgggtg gagaggctat
      361 tcggctatga ctgggcacaa cagacaatcg gctgctctga tgccgccgtg ttccggctgt
      421 cagcgcaggg gcgcccggtt ctttttgtca agaccgacct gtccggtgcc ctgaatgaac
      481 tgcaggacga ggcagcgcgg ctatcgtggc tggccacgac gggcgttcct tgcgcagctg
      541 tgctcgacgt tgtcactgaa gcgggaaggg actggctgct attgggcgaa gtgccggggc
      601 aggatctcct gtcatcccac cttgctcctg ccgagaaagt atccatcatg gctgatgcaa
      661 tgcggcggct gcatacgctt gatccggcta cctgcccatt cgaccaccaa gcgaaacatc
      721 gcatcgagcg agcacgtact cggatggaag ccggtcttgt cgatcaggat gatctggacg
      781 aagagcatca ggggctcgcg ccagccgaac tgttcgccag gctcaaggcg cgcatgcccg
      841 acggcgagga tctcgtcgtg acccatggcg atgcctgctt gccgaatatc atggtggaaa
      901 atggccgctt ttctggattc atcgactgtg gccggctggg tgtggcggac cgctatcagg
      961 acatagcgtt ggctacccgt gatattgctg aagagcttgg cggcgaatgg gctgaccgct
     1021 tcctcgtgct ttacggtatc gccgctcccg attcgcagcg catcgccttc tatcgccttc
     1081 ttgacgagtt cttctgaacc ggtaatatta ttgaagcatt tatcagggtt attgtctcat
     1141 gagcggatac atatttgaat gtatttagaa aaataaacaa ataggggttc cgcgcacatt
     1201 tccccgaaaa gtgccacctg acgtctaaga aaccattatt atcatgacat taacctataa
     1261 aaataggcgt atcacgaggc cctttcgtct cgcgcgtttc ggtgatgacg gtgaaaacct
     1321 ctgacacatg cagctcccgg agacggtcac agcttgtctg taagcggatg ccgggagcag
```

1381 acaagcccgt cagggcgcgt cagcgggtgt tggcgggtgt cggggctggc ttaactatgc
1441 ggcatcagag cagattgtac tgagagtgca ccaattaaat gccatttcat tacctctttc
1501 tccgcacccg acatagatct tgatgttcct attgcgtctt ctcttacaaa tggctgaata
1561 acgatgtttc tatccagtta gtgggataga tagattgctg atagtggcac ttatattgaa
1621 tgtgaatgcg aattcagtac tgctagcaag agctcgatct agaggtaccc gttacataac
1681 ttacggtaaa tggcccgcct ggctgaccgc ccaacgaccc ccgcccattg acgtcaatag
1741 taacgccaat agggactttc cattgacgtc aatgggtgga gtatttacgg taaactgccc
1801 acttggcagt acatcaagtg tatcatatgc caagtacgcc ccctattgac gtcaatgacg
1861 gtaaatggcc cgcctggcat tgtgcccagt acatgacctt atgggacttt cctacttggc
1921 agtacatcta cgtattagtc atcgctatta ccatggtcga ggtgagcccc acgttctgct
1981 tcactctccc catctccccc ccctccccac ccccaatttt gtatttattt atttttaat
2041 tattttgtgc agcgatgggg gcgggggggg gggggggcg cgcgccaggc ggggcggggc
2101 ggggcgaggg gcggggcggg gcgaggcgga gaggtgcggc ggcagccaat cagagcggcg
2161 cgctccgaaa gtttcctttt atggcgaggc ggcggcggcg gcggccctat aaaaagcgaa
2221 gcgcgcggcg ggcgggagtc gctgcgacgc tgccttcgcc ccgtgccccg ctccgccgcc
2281 gcctcgcgcc gcccgccccg gctctgactg accgcgttac tcccacaggt gagcgggcgg
2341 gacggccctt ctcctccggg ctgtaattag ctgagcaaga ggtaagggtt taagggatgg
2401 ttggttggtg gggtattaat gtttaattac ctggagcacc tgcctgaaat cactttttt
2461 caggttggac cggtgccacc atggactata aggaccacga cggagactac aaggatcatg
2521 atattgatta caaagacgat gacgataaga tggccccaaa gaagaagcgg aaggtcggta
2581 tccacggagt cccagcagcc gacaagaagt acagcatcgg cctggacatc ggcaccaact
2641 ctgtgggctg ggccgtgatc accgacgagt acaaggtgcc cagcaagaaa ttcaaggtgc
2701 tgggcaacac cgaccggcac agcatcaaga agaacctgat cggagccctg ctgttcgaca
2761 gcggcgaaac agccgaggcc acccggctga agagaaccgc cagaagaaga tacaccagac
2821 ggaagaaccg gatctgctat ctgcaagaga tcttcagcaa cgagatggcc aaggtggacg
2881 acagcttctt ccacagactg gaagagtcct tcctggtgga agaggataag aagcacgagc
2941 ggcaccccat cttcggcaac atcgtggacg aggtggccta ccacgagaag taccccacca
3001 tctaccacct gagaaagaaa ctggtggaca gcaccgacaa ggccgacctg cggctgatct
3061 atctggccct ggcccacatg atcaagttcc ggggccactt cctgatcgag ggcgacctga
3121 accccgacaa cagcgacgtg gacaagctgt tcatccagct ggtgcagacc tacaaccagc
3181 tgttcgagga aaaccccatc aacgccagcg gcgtggacgc caaggccatc ctgtctgcca
3241 gactgagcaa gagcagacgg ctggaaaatc tgatcgccca gctgcccggc gagaagaaga
3301 atggcctgtt cggaaacctg attgccctga gcctgggcct gacccccaac ttcaagagca
3361 acttcgacct ggccgaggat gccaaactgc agctgagcaa ggacacctac gacgacgacc
3421 tggacaacct gctggcccag atcggcgacc agtacgccga cctgtttctg gccgccaaga
3481 acctgtccga cgccatcctg ctgagcgaca tcctgagagt gaacaccgag atcaccaagg
3541 ccccctgag cgcctctatg atcaagagat acgacgagca ccaccaggac ctgaccctgc
3601 tgaaagctct cgtgcggcag cagctgcctg agaagtacaa agagattttc ttcgaccaga
3661 gcaagaacgg ctacgccggc tacattgacg gcggagccag ccaggaagag ttctacaagt
3721 tcatcaagcc catcctggaa aagatggacg gcaccgagga actgctcgtg aagctgaaca
3781 gagaggacct gctgcggaag cagcggacct tcgacaacgg cagcatcccc caccagatcc
3841 acctgggaga gctgcacgcc attctgcggc ggcaggaaga tttttaccca ttcctgaagg
3901 acaaccggga aaagatcgag aagatcctga ccttccgcat cccctactac gtgggccctc
3961 tggccagggg aaacagcaga ttcgcctgga tgaccagaaa gagcgaggaa accatcaccc
4021 cctggaactt cgaggaagtg gtggacaagg gcgcttccgc ccagagcttc atcgagcgga
4081 tgaccaactt cgataagaac ctgcccaacg agaaggtgct gcccaagcac agcctgctgt
4141 acgagtactt caccgtgtat aacgagctga ccaaagtgaa atacgtgacc gagggaatga
4201 gaaagcccgc cttcctgagc ggcgagcaga aaaaggccat cgtggacctg ctgttcaaga
4261 ccaaccggaa agtgaccgtg aagcagctga aagaggacta cttcaagaaa atcgagtgct
4321 tcgactccgt ggaaatctcc ggcgtggaag atcggttcaa cgcctccctg ggcacatacc
4381 acgatctgct gaaaattatc aaggacaagg acttcctgga caatgaggaa aacgaggaca
4441 ttctggaaga tatcgtgctg accctgacac tgtttgagga cagagagatg atcgaggaac
4501 ggctgaaaac ctatgcccac ctgttcgacg acaaagtgat gaagcagctg aagcggcgga
4561 gatacaccgg ctggggcagg ctgagccgga agctgatcaa cggcatccgg gacaagcagt
4621 ccggcaagac aatcctggat ttcctgaagt ccgacggctt cgccaacaga aacttcatgc
4681 agctgatcca cgacgacagc ctgaccttta aagaggacat ccagaaagcc caggtgtccg
4741 gccagggcga tagcctgcac gagcacattg ccaatctggc cggcagcccc gccattaaga

4801 agggcatcct gcagacagtg aaggtggtgg acgagctcgt gaaagtgatg ggccggcaca
4861 agcccgagaa catcgtgatc gaaatggcca gagagaacca gaccacccag aagggacaga
4921 agaacagccg cgagagaatg aagcggatcg aagagggcat caaagagctg ggcagccaga
4981 tcctgaaaga acaccccgtg gaaaacaccc agctgcagaa cgagaagctg tacctgtact
5041 acctgcagaa tgggcgggat atgtacgtgg accaggaact ggacatcaac cggctgtccg
5101 actacgatgt ggaccatatc gtgcctcaga gctttctgaa ggacgactcc atcgacaaca
5161 aggtgctgac cagaagcgac aagaaccggg gcaagagcga caacgtgccc tccgaagagg
5221 tcgtgaagaa gatgaagaac tactggcggc agctgctgaa cgccaagctg attacccaga
5281 gaaagttcga caatctgacc aaggccgaga gaggcggcct gagcgaactg gataaggccg
5341 gcttcatcaa gagacagctg gtggaaaccc ggcagatcac aaagcacgtg gcacagatcc
5401 tggactcccg gatgaacact aagtacgacg agaatgacaa gctgatccgg gaagtgaaag
5461 tgatcaccct gaagtccaag ctggtgtccg atttccggaa ggatttccag ttttacaaag
5521 tgcgcgagat caacaactac caccacgccc acgacgccta cctgaacgcc gtcgtgggaa
5581 ccgccctgat caaaaagtac cctaagctgg aaagcgagtt cgtgtacggc gactacaagg
5641 tgtacgacgt gcggaagatg atcgccaaga gcgagcagga aatcggcaag gctaccgcca
5701 agtacttctt ctacagcaac atcatgaact tttcaagac cgagattacc ctggccaacg
5761 gcgagatccg gaagcggcct ctgatcgaga caaacggcga aaccggggag atcgtgtggg
5821 ataagggccg ggattttgcc accgtgcgga aagtgctgag catgccccaa gtgaatatcg
5881 tgaaaaagac cgaggtgcag acaggcggct tcagcaaaga gtctatcctg cccaagagga
5941 acagcgataa gctgatcgcc agaaagaagg actgggaccc taagaagtac ggcggcttcg
6001 acagccccac cgtggcctat tctgtgctgg tggtggccaa agtggaaaag ggcaagtcca
6061 agaaactgaa gagtgtgaaa gagctgctgg ggatcaccat catggaaaga agcagcttcg
6121 agaagaatcc catcgacttt ctggaagcca agggctacaa agaagtgaaa aaggacctga
6181 tcatcaagct gcctaagtac tccctgttcg agctggaaaa cggccggaag agaatgctgg
6241 cctctgccgg cgaactgcag aagggaaacg aactggccct gccctccaaa tatgtgaact
6301 tcctgtacct ggccagccac tatgagaagc tgaagggctc ccccgaggat aatgagcaga
6361 aacagctgtt tgtggaacag cacaagcact acctggacga gatcatcgag cagatcagcg
6421 agttctccaa gagagtgatc ctggccgacg ctaatctgga caaagtgctg tccgcctaca
6481 acaagcaccg ggataagccc atcagagagc aggccgagaa tatcatccac ctgtttaccc
6541 tgaccaatct gggagcccct gccgccttca agtactttga caccaccatc gaccggaaga
6601 ggtacaccag caccaaagag gtgctggacg ccaccctgat ccaccagagc atcaccggcc
6661 tgtacgagac acggatcgac ctgtctcagc tgggaggcga caaaaggccg gcggccacga
6721 aaaaggccgg ccaggcaaaa aagaaaaagt aagaattcct agagctcgct gatcagcctc
6781 gactgtgcct tctagttgcc agccatctgt tgtttgcccc tcccccgtgc cttccttgac
6841 cctggaaggt gccactccca ctgtcctttc ctaataaaat gaggaaattg catcgcattg
6901 tctgagtagg tgtcattcta ttctgggggg tggggtgggg caggacagca agggggagga
6961 ttgggaagag aatagcaggc atgctgggga gcggccatcg atcagggtac cttggatccg
7021 atctagactt aattaaggtc gactactgca gttgcatgcc cccgggtcgc gagtactcac
7081 tacgtgccta gcgagggcct atttcccatg attccttcat atttgcatat acgatacaag
7141 gctgttagag agataattgg aattaatttg actgtaaaca caaagatatt agtacaaaat
7201 acgtgacgta gaaagtaata atttcttggg tagtttgcag ttttaaaatt atgttttaaa
7261 atggactatc atatgcttac cgtaacttga aagtatttcg atttcttggc tttatatatc
7321 ttgtggaaag gacgaaacac cggagacccg actagtaggg tctctgtttt agagctagaa
7381 atagcaagtt aaaataaggc tagtccgtta tcaacttgaa aaagtggcac cgagtcggtg
7441 ctttttgtt ttagagctag aaatagcaag ttaaaataag gctagtccgt ttttagcgcg
7501 tgcgccaatt ctgcaggcta ggtggaggaa taataatgtc tttcggagag atggtgagtg
7561 gttgaaagcc ggtctttgta acatcacaag gagcaggaca gtgctgatgc agtaagagta
7621 taacgatgtc atagatcttt cgctacctta ggaccgttat agttacattt aattcctgtg
7681 tgaaattgtt atccgctcac aattccacac aacatacgag ccggaagcat aaagtgtaaa
7741 gcctggggtg cctaatgagt gagctaactc acattaattg cgttgcgctc actgcccgct
7801 ttccagtcgg gaaacctgtc gtgccagctg cattaatgaa tcggccaacg cgcggggaga
7861 ggcggtttgc gtattgggcg ctcttccgct tcctcgctca ctgactcgct gcgctcggtc
7921 gttcggctgc ggcgagcggt atcagctcac tcaaaggcgg taatacggtt atccacagaa
7981 tcaggggata acgcaggaaa gaacatgtga gcaaaaggcc agcaaaaggc caggaaccgt
8041 aaaaaggccg cgttgctggc gtttttccat aggctccgcc cccctgacga gcatcacaaa
8101 aatcgacgct caagtcagag gtggcgaaac ccgacaggac tataaagata ccaggcgttt
8161 ccccctggaa gctccctcgt gcgctctcct gttccgaccc tgccgcttac cggatacctg

8221 tccgcctttc tcccttcggg aagcgtggcg ctttctcata gctcacgctg taggtatctc
8281 agttcggtgt aggtcgttcg ctccaagctg ggctgtgtgc acgaaccccc cgttcagccc
8341 gaccgctgcg ccttatccgg taactatcgt cttgagtcca acccggtaag acacgactta
8401 tcgccactgg cagcagccac tggtaacagg attagcagag cgaggtatgt aggcggtgct
8461 acagagttct tgaagtggtg gcctaactac ggctacacta gaaggacagt atttggtatc
8521 tgcgctctgc tgaagccagt taccttcgga aaaagagttg gtagctcttg atccggcaaa
8581 caaaccaccg ctggtagcgg tggttttttt gtttgcaagc agcagattac gcgcagaaaa
8641 aaaggatctc a//

pBHCA-eGFP-HOM-CCBD-AMP-HOM

```
LOCUS       Exported               36675 bp ds-DNA     circular UNK 07-JUN-2016
DEFINITION  .
ACCESSION   .
VERSION     .
KEYWORDS    pBHCA-eGFP-NSH-ccdb-amp
SOURCE      natural DNA sequence
  ORGANISM  unspecified
REFERENCE   1  (bases 1 to 36675)
  AUTHORS   .
  TITLE     Direct Submission
  JOURNAL   Exported Donnerstag, 9. Jun 2016 from SnapGene 2.5.0
            http://www.snapgene.com
COMMENT     This file is created by VectorDesigner
FEATURES             Location/Qualifiers
     source          1..36675
                     /organism="unspecified"
                     /mol_type="genomic DNA"
     misc_feature    1..7959
                     /label="Fragment 3 (114914-106508 NC_000023)"
                     /note="Fragment 3"
     CDS             complement(664..681)
                     /codon_start=1
                     /product="6xHis affinity tag"
                     /note="6xHis"
                     /translation="HHHHHH"
     CDS             complement(825..842)
                     /codon_start=1
                     /product="6xHis affinity tag"
                     /note="6xHis"
                     /translation="HHHHHH"
     misc_feature    7987..8102
                     /note="left homology arm"
     promoter        8168..8259
                     /gene="bla"
                     /note="AmpR promoter"
     CDS             8260..9120
                     /codon_start=1
                     /gene="bla"
                     /product="beta-lactamase"
                     /note="AmpR"
                     /note="confers resistance to ampicillin, carbenicillin, and
                     related antibiotics"
                     /translation="MSIQHFRVALIPFFAAFCLPVFAHPETLVKVKDAEDQLGARVGYI
                     ELDLNSGKILESFRPEERFPMMSTFKVLLCGAVLSRVDAGQEQLGRRIHYSQNDLVEYS
                     PVTEKHLTDGMTVRELCSAAITMSDNTAANLLLTTIGGPKELTAFLHNMGDHVTRLDRW
                     EPELNEAIPNDERDTTMPAAMATTLRKLLTGELLTLASRQQLIDWMEADKVAGPLLRSA
                     LPAGWFIADKSGAGERGSRGIIAALGPDGKPSRIVVIYTTGSQATMDERNRQIAEIGAS
                     LIKHW"
     CDS             9169..9474
                     /codon_start=1
                     /gene="ccdB"
                     /product="CcdB, a bacterial toxin that poisons DNA gyrase"
                     /note="ccdB"
                     /note="Plasmids containing the ccdB gene cannot be
                     propagated in standard E. coli strains."
```

/translation="MQFKVYTYKRESRYRLFVDVQSDIIDTPGRRMVIPLASARLLSDK
VSRELYPVVHIGDESWRMMTTDMASVPVSVIGEEVADLSHRENDIKNAINLMFWGI"
misc_feature 9545..9656
/note="right homology arm"
misc_feature 9695..18733
/label="Fragment 2 (129441-119715 of NC_000023)"
/note="Fragment 2"
primer_bind 18441..18462
/label="mhBHCA-Blank 5'"
/note="mhBHCA-Blank 5'"
primer_bind 18915..18934
/note="primer BHCA-18365seq5'"
polyA_signal 19025..19249
/note="bGH poly(A) signal"
/note="bovine growth hormone polyadenylation signal"
primer_bind 19026..19045
/label="eGFP det 5'"
/note="eGFP det 5'"
polyA_signal complement(19041..19255)
/label="BGH pA-signal"
/note="BGH pA-signal"
CDS complement(19301..20020)
/codon_start=1
/label="eGFP"
/note="eGFP"
/translation="MVSKGEELFTGVVPILVELDGDVNGHKFSVSGEGEGDATYGKLTL
KFICTTGKLPVPWPTLVTTLTYGVQCFSRYPDHMKQHDFFKSAMPEGYVQERTIFFKDD
GNYKTRAEVKFEGDTLVNRIELKGIDFKEDGNILGHKLEYNYNSHNVYIMADKQKNGIK
VNFKIRHNIEDGSVQLADHYQQNTPIGDGPVLLPDNHYLSTQSALSKDPNEKRDHMVLL
EFVTAAGITLGMDELYK"
promoter complement(20087..20105)
/note="T7 promoter"
/note="promoter for bacteriophage T7 RNA polymerase"
promoter complement(20104..20759)
/label="CMV promotor"
/note="CMV promotor"
enhancer 20354..20733
/note="CMV enhancer"
/note="human cytomegalovirus immediate early enhancer"
primer_bind complement(20751..20770)
/label="eGFP det 3'"
/note="eGFP det 3'"
misc_feature 20825..29336
/label="Fragment 1 (155517-147010 NC_000023)"
/note="Fragment 1"
primer_bind complement(20833..20882)
/label="mhBHCA-nested 3'"
/note="mhBHCA-nested 3'"
misc_feature 29555..29751
/label="3' ITR"
/note="3' ITR"
repeat_region 29650..29752
/note="ITR"
/note="inverted terminal repeat of human adenovirus
serotype 5"
primer_bind 29733..29782
/label="primer BAC-bb-for"

/note="primer BAC-bb-for"
misc_feature 29733..29762
/label="HR for pHCA-eGFP"
/note="HR for pHCA-eGFP"
misc_feature 29763..29782
/label="HR for BAC-backbone"
/note="HR for BAC-backbone"
CDS complement(29921..30580)
/codon_start=1
/gene="cat"
/product="chloramphenicol acetyltransferase"
/note="CmR"
/note="confers resistance to chloramphenicol"
/translation="MEKKITGYTTVDISQWHRKEHFEAFQSVAQCTYNQTVQLDITAFL
KTVKKNKHKFYPAFIHILARLMNAHPEFRMAMKDGELVIWDSVHPCYTVFHEQTETFSS
LWSEYHDDFRQFLHIYSQDVACYGENLAYFPKGFIENMFFVSANPWVSFTSFDLNVANM
DNFFAPVFTMGKYYTQGDKVLMPLAIQVHHAVCDGFHVGRMLNELQQYCDEWQGGA"
promoter complement(30581..30683)
/note="cat promoter"
/note="promoter of the E. coli cat gene"
rep_origin 31607..31826
/note="ori2"
/note="secondary origin of replication for the bacterial F
plasmid; also known as oriS"
CDS 31917..32672
/codon_start=1
/gene="repE"
/product="replication initiation protein for the bacterial
F plasmid"
/note="repE"
/translation="MAETAVINHKKRKNSPRIVQSNDLTEAAYSLSRDQKRMLYLFVDQ
IRKSDGTLQEHDGICEIHVAKYAEIFGLTSAEASKDIRQALKSFAGKEVVFYRPEEDAG
DEKGYESFPWFIKRAHSPSRGLYSVHINPYLIPFFIGLQNRFTQFRLSETKEITNPYAM
RLYESLCQYRKPDGSGIVSLKIDWIIERYQLPQSYQRMPDFRRRFLQVCVNEINSRTPM
RLSYIEKKKGRQTTHIVFSFRDITSMTTG"
misc_feature 32675..32925
/gene="incC"
/note="incC"
/note="incompatibility region of the bacterial F plasmid"
CDS 33251..34426
/codon_start=1
/gene="sopA"
/product="partitioning protein for the bacterial F plasmid"
/note="sopA"
/translation="MFRMKLMETLNQCINAGHEMTKAIAIAQFNDDSPEARKITRRWRI
GEAADLVGVSSQAIRDAEKAGRLPHPDMEIRGRVEQRVGYTIEQINHMRDVFGTRLRRA
EDVFPPVIGVAAHKGGVYKTSVSVHLAQDLALKGLRVLLVEGNDPQGTASMYHGWVPDL
HIHAEDTLLPFYLGEKDDVTYAIKPTCWPGLDIIPSCLALHRIETELMGKFDEGKLPTD
PHLMLRLAIETVAHDYDVIVIDSAPNLGIGTINVVCAADVLIVPTPAELFDYTSALQFF
DMLRDLLKNVDLKGFEPDVRILLTKYSNSNGSQSPWMEEQIRDAWGSMVLKNVVRETDE
VGKGQIRMRTVFEQAIDQRSSTGAWRNALSIWEPVCNEIFDRLIKPRWEIR"
CDS 34426..35397
/codon_start=1
/gene="sopB"
/product="partitioning protein for the bacterial F plasmid"
/note="sopB"
/translation="MKRAPVIPKHTLNTQPVEDTSLSTPAAPMVDSLIARVGVMARGNA

ITLPVCGRDVKFTLEVLRGDSVEKTSRVWSGNERDQELLTEDALDDLIPSFLLTGQQTP
AFGRRVSGVIEIADGSRRRKAAALTESDYRVLVGELDDEQMAALSRLGNDYRPTSAYER
GQRYASRLQNEFAGNISALADAENISRKIITRCINTAKLPKSVVALFSHPGELSARSGD
ALQKAFTDKEELLKQQASNLHEQKKAGVIFEAEEVITLLTSVLKTSSASRTSLSSRHQF
APGATVLYKGDKMVLNLDRSRVPTECIEKIEAILKELEKPAP"
misc_feature 35470..35943
/gene="sopC"
/note="sopC"
/note="centromere-like partitioning region of the bacterial
F plasmid"
primer_bind 36120..36169
/label="primer wt/Hv-BAC-Zeo"
/note="primer wt/Hv-BAC-Zeo"
primer_bind complement(36161..36210)
/label="primer BAC-bb-rev"
/note="primer BAC-bb-rev"
misc_feature complement(36161..36180)
/label="HR for BAC-backbone"
/note="HR for BAC-backbone"
misc_feature complement(36181..36210)
/label="HR for pHCA-eGFP"
/note="HR for pHCA-eGFP"
promoter 36182..36200
/note="T7 promoter"
/note="promoter for bacteriophage T7 RNA polymerase"
repeat_region 36231..36333
/note="ITR"
/note="inverted terminal repeat of human adenovirus
serotype 5"
misc_feature 36232..36666
/label="5'ITR & packaging signal"
/note="5'ITR & packaging signal"
misc_feature complement(36409..36438)
/label="HR 2"
/note="HR 2"
misc_signal 36421..36571
/note="Ad5 Psi"
/note="packaging signal for adenovirus serotype 5"
ORIGIN
1 ggccgaagga ttacatgagc ttagaaatgt aattagcata gtgtgtggca tagtgtagat
61 accaaataaa tatgatctct ccttctactc ttgaaaatgc aaacacattc ttggtggtcc
121 taaaatagcc tgtaacatgg tttactcagc agcatttgct attcaaggca gatctgcctt
181 tagtcattgg ctgcgctcct gaacagctgt gtgaaaggct aacttttgta aaccaaatca
241 aaataaaatg cagcaaaaat ttgtcactga aaggaaatcc tcagtatatc cttttatgaa
301 atgaaagatc cctcatccaa acttaacttt tttaaaagtg cgcatttgga gatatagccc
361 tttcttatga atcctaattc aattttggcc ataaacacac gttgatgttc cccaccccaa
421 agcacatagc aacaagagta ggttctatat tgaaaataat gacaatttaa aaacatgtac
481 ttatttcact gtatgtggac agtgtctatg attgcatcat gaagtgtcat ataaccatgt
541 acgtgtacat gagagagaga tagagagaga agtggtaggg tggtggtggt agaggggatg
601 gcgatagtaa tcatggtaat ggtagaggtg atggaggtgg taatgacgga ggtaagggtg
661 gtagtgatga tggtggtggt ggtaatggtg gtggatgtgg tggtggcaat tgggatggtg
721 ggatggtggt agccatggtg atggtggtaa tggtgttgat ttaaagggtg gtggtagtga
781 aggtgagggt agtggtggtg gaggtggtgg tgctggtagc aatagtgatg gtggtgatgg
841 tgttgatgag ggtgttggga tcagggtgag ttcccacagt atatttcatt cttgttgtac
901 cactctgtca acagcaccac tgactgggac agaggaagaa ggcacactct gaatgtgtta
961 ttaacagaaa cctcaaaaca gtctgtctcc ttgtagtcat tcaaaattat ctttttctta
1021 cctggaaaac tgaaactgaa ttaccgggaa aaacacagga gatttttgtt tgttaatatg

1081 ctgccaataa agtaatttta tgtcaaattt aactacagga aagggcaagg cattttctaa
1141 gttccttaga tgtcatgtgg ctaaaaaaaa caaaaggatg gacagcagtt agatactgta
1201 cacttagctg tttgaagcca tatattcaga aagcagatgt tgggagttgg tgtttgagga
1261 ctgatttcct ggaggtattt tatataggcc aagttcattg ttctaaactc taagggcttg
1321 acttgaggga ggaaaagagg caagaacatg tttagttttg ctgacagcat cacatgggca
1381 gccctaaggc tagacaactt tagggcctga agcttattct aggaaagaag cacctacaga
1441 gtggcactgg gctcccctcc actatagaga tgaagtcata tgacagtaaa gggcaggcag
1501 ggctgcctag ggggcccaga actgacactt ccattagaat gagcacaggc cagggagaga
1561 agtggggaac cagagagaag gagctggaat tctagtagga caaacggtaa gtgaacaaca
1621 agaacaagtt aagagtgtgt gcagtattct ttcaaagact gaaaaaatag tgatgtgata
1681 gaatggcagg tggctctgag caggccagga gaaggactgg gggcagagca tcccaggcag
1741 gagggcagca agtgggaagg ccctggggtg gggcttttgg actgttccag tgacgggcag
1801 gcagccagtg tgcctgtcac acaatgcacc agggaagtag tcgtgaattt gcagagggtc
1861 ttgcaggcta tgggaaaggg attggattgt attttgtttg tagggaagcc atcgggggac
1921 ttaagcagag gaaggattgg cttcatctct ttgaaaaagt tctctctgga tgctgatggg
1981 aggagaaatg gaaggaaaag aaacactttt aggggcaaga acttttgaga agggtggaat
2041 tgggagtgtg gagttggggc cagctttggc acaggagggg aagctaaaca cgtggccgca
2101 tgagggcctg taattctacc tgaaatgggt accatttgtt agggtaaaca aatgaaccaa
2161 atgcccagtg atacagacca agtgttggca aacttcttct gtgatggccc aggtagtaaa
2221 tgtctcaggc ttcgcaggcc atgtggtctc tgttgaagct ctgtgtagta gacaatatgt
2281 taatgactgg gcgtgactgt gtgctaataa aagtttattt acaaaaacag cccgtgggct
2341 ggatttagct cacaggctgt agtttgccaa cctctgacct agagcatgaa ctgagcatct
2401 tcttggaggg aaataagttc tttccaagtt gccctcctca cattgcaggg ggccatgtag
2461 gcccattatt cacagaagag tgggtgggca acctttctgg agcagaaaaa cgtaaagatt
2521 tcttccgtag tgcaagtaag gtgaccattt ctaaaccgtg caagtgatcc agcagtccca
2581 aaagttgttt cacttctcat tgtgcgcccg ttctcaggtg ctccgaagct tccagtcctt
2641 tgtagggaca tggatgaaat tggaaatcat cattctcagt aaactatcgc aagaacaaaa
2701 aaccaaacac cgcatattct cactcatagg tgggaattga acaatgagat cacatggaca
2761 cgggaagggg aacatcacat tctggggact gttgtggggt ggggggaggg gggagggata
2821 gcattgggag atatacctaa tgctagatga caagttagtg ggtgcagcgc accagtgtgg
2881 cacatgtata catatgtaac taacctgcac aatgtgcaca tgtaccctaa aacttaaagt
2941 ataataataa aagaaaaaaa aaaagagagg agagaaacat catcccctcc aggataccct
3001 tgggccttgt tcttatagtc ttgtacattg ttgaacaatt tgcatgggct agtggattaa
3061 agcacaccct ccaccctcag gccctcaagg gtctctatga taatacagtc tcaccttcta
3121 ccctttccat caccatccta ggtgctatgg ccaaccttga ggctgccatg ttaggtctat
3181 gcatttccca cctccaccac ataactctct gaaggccagg tagtttccta ttcatcttgg
3241 taaccccaaa gcctcgtgac agggctcagc tggcatctgc ggatgtgaat gaaccattgg
3301 agaaaatggt actctgcaaa taactctgtt attttcccat ttcctgtgta aggcctagag
3361 acaatgactt tttaattgca ccccttcccc tctgtatgac actggccttc tcttgtgtcc
3421 agcaatgtgg gtggcctaga tgatttctaa gggacttctg gccaagatga acagcagctg
3481 catcttactg agcatttact atgtgccata tactcagcca cagctctagg ggcatagaag
3541 caggagctct cagggtcagg gcagtgagtg agcaagcgag cacctatgcc agccctgcct
3601 ctggatgggg acttgagagg gtgatggaag cctgcagcac tggagggagg cagacaaaga
3661 caggcctgtg ctgagggggc ccggagcaag agagagggag gcaatgacag cagagacatg
3721 cctgcgcctt gggtttgagt gcccagtggt caaatccact tccctgtggc tgatgcttgc
3781 ctttctaact ttggaattta ggggttggag atctggtgag aaggtaggag ggagatgagg
3841 aggagaaggg aaaggcagga aggaagggga gggaaaggaa aagcaaaagg ggaggaggaa
3901 ggtttccaac aaattattct atatcaactg cggaaatcaa aatttgttgc ccaaatctta
3961 gaagctcatg tccctcctcc ccagaagtct ggaatgcagc actccagggg tagcttataa
4021 cccaaatatc tatctgtaaa aagagaaaca ttgggctttc gagctgtgga ttctcagtaa
4081 aagcaagagg cctcagccta cacaggccag cccagagttt gaggaacccc aggcccacac
4141 ccacagggct ggcccctggg tctgcatact ccctagaaat gtgcacactt ctgagcctca
4201 actctgtcct ggagtctaac agcatccctc tccttcctgg ggcagttcca cctccagaaa
4261 cctgttacct tgggccttat gtcaaggaaa ctgtgggaaa gagctaggca ggaatgcaga
4321 tgaggccagc atgggctcct aaaagtttag aaataggcag tgtcatgctc ccaggtgcct
4381 gcataaacca gctgaaaaat ggagctcccc tcaccagcac tctcccttca aacagactgt
4441 gatttgcagg tcactggttt accaagccag gctacccagg caggacccag atgccaagcc

4501 cagtggtgtc ctgcaagctg agcagtgctc agttcttgca aaaaaaggtc tgtgtgaagg
4561 caaggcctct gcctggcttc tcaccccagt tgggtgtctg gaacaggaag gagcccttac
4621 tgcagaaaaa ggaggaggga gcaaagggag cgaacagctg cgtgctccat ggggaggatc
4681 cccaaagtag aaaggcgcat acacactgca gcccttgacc cagaatgctc acagctacat
4741 tacagattca ggtctcctca gtgtagtggg gctgctgatg agactgtggc atcctcaggg
4801 gtcaggacac acattttcca tcactcttct gatggcaaaa aacctctgag ccaatgccaa
4861 cctctgatca ttaaaaaaaa gtgctcacag cagtgtgtgg tttaggatca tgccctgtgt
4921 ggtttggaac acgtgcacaa ccacaccttg ttcatcacca tcccagaaac cctgacgcag
4981 gcaaagagca gagttattaa ccctacttta ctgatgtgga tactgaggcc cagaggctca
5041 tgcaagttat caataagtgg cagggacagt tgcctctaga ttaactagcc cctaggatca
5101 cctgggtctt ggaaggggac ccataaacat gagctcccct ctcttggggc cagatttgca
5161 cctgtgccgc gccttcagcc tgcatgaagt aggggctgct ggcaaagact caaagctgta
5221 aatctgggtt ttctcttgag gcttctaagg gagctgtttc gacaactcac tctgttccca
5281 gctggctgcc cctgcatagg gttttaaagc agcctagctt tctgccaggc ttggcagtgg
5341 acaacgctgg tcagaacatc ccagagagct accagaatga agtaagtttg cttctactct
5401 ttacctgttt atgggctgtc tctgccactg gaatgaaagg cactgagaac agtgcctggc
5461 ctgcagaagg ccctggaaat acctgagctc ctaatctggg aataggagta ggaagagctt
5521 tggaggcagg gcacctgagt ttgagatcta caacttcctg cctgtgtgac attgggaaag
5581 tctccatcct ttctgagcct cagtctccac cctggggaag tggaaatatc aatctctgtg
5641 acacagaagc aaatgagcga atgtgcacaa agtaccttgc acaagagaga cgctcaaaca
5701 cttgcctcca ggtttcaccg agaactacag agtaagatag atttgttccc agtggaggaa
5761 gcctgggaat aatttgcccc tagactatga attcctgggg ctcaagatcg agcacagggc
5821 caggcacaca gaagggaccc tggaaatgtg gcaggaggcc agagatagac aggcccttag
5881 agctcatacc catgccctct gacctcaaga agaaagaaac ctgctcaaaa tctcacaaag
5941 agcttgttcc aaccctgaat cgagtctgag gactccttcc tgagtccagc actttttctg
6001 caagaagtat atgcctccaa agctgatggg cgcaaatctt gaaccccgtc acataaacac
6061 aaagggagga ggtgactaga gctcctccta ctggatatgt ctaaggtcac cagtctaaag
6121 aaaagggatg gatagaatga ggccagtatt tttgcagcca tccaaatgtc cacatacgct
6181 gttacactga gggctcctct ctcccccgtc ttcagcccta cttgcattta gaggtgagaa
6241 agatatgggc tgaggggttg tttttcatcg tattgtagat ggaaagcaca ctgcccttgg
6301 ggccatccaa atgtggacct tgatgtagca ccccaccttc tggatggcca tccttctgaa
6361 agtcactgaa tttctcagac tttattctct ttatccataa agaaggagaa taataataat
6421 ccccccaccc tgcccaacca ctgactggtt gggaagctca gaagaaatac tgggcacggc
6481 atcccattgt aatctataga gtgagtcgct tcttaatatt aaatggctga acacagaaga
6541 tgtgcaaaaa gtactgtgtc cccttcctcc tccaactgaa catttcatgc cctttgcacc
6601 ctcattttgt ctaggagctg ccttatgaag ggaataggta cctgctccga gctggaggaa
6661 tctttgccac ttatggtggg gtatggactg agacagagat ggcatgtgac atgcgcactg
6721 agtctcaact ccatgcaggc tctggagcac tctcaaattg gagtactaat gccttttaaa
6781 ttctcacact agcaatcctt tgacctactg atctagggat ctagggaaag aatcgtgatc
6841 ttaacttcaa agggaaggac aaaatgttct gcctcctgtt aaaactccat acactaagtg
6901 cagagactgg atgccttatt aaccttgggt agatgcccaa atgttcaaaa ggtcaaactc
6961 ttctgttccc cagatcgcca gagtcattaa ccagtcacac tattaaatga atgaacagat
7021 gctgaaaagg tacttgcatt actgagattt cttatggtga tggcccctgc ctgatatgta
7081 ttcagcattt tgtagttttc aatgtgcatt agagtatagt ggtgatgaca ttggcctctg
7141 agtttgccac ttcttatatc tgtgactttg gtcaaattgc ttaatctctc tgagtctcgg
7201 tttcctggag ataataatag cttcttcttc ccagggttat catgaggatt acaggagata
7261 atgccccaaa aatgcttagt aaagtgccta gcacctagtc aatgctgaat taaaggtggt
7321 tattcttact tttcgttcat ttgaactttg ttctcaggga gggcaaagga tagacaaagc
7381 cccatagcta gtgaggagta gctgcaagac tagaacccag gtgttctgag ccctagtctt
7441 aggccaagaa caactgttac gtgagatgca cgttttcctt caagggagct cacaattatt
7501 tccatgtaaa ttcaaggact gctaaaagag aactctcctc tgggactgat atcattttat
7561 ttcaagattg atttgaaaca tgttttttgt ttgtttgttt gttttctagg aaagaacaag
7621 agaaccagtc aagctgaatg cctgaagcaa atctctgtta gcgatgtttt caggatgagg
7681 gagagtggtg caagaaacgt gcttccagat gcacatggtt tcctgggact agggttcagg
7741 gtgtcatccc tgggtgttat taagtgtcag aaggagagca aacaaggaa acatctgaga
7801 tccagctaag gctacaccct ggaaatgcaa gcccagctct tgcaaaggac ctcctttggc
7861 cactcacctt ccaggcctta caataacttg tttggactgc aggtttcttg gtggactcac

7921 aggccattct gcttttattt ggtcaacctc agttcacaat aactataacg gtcctaaggt
7981 agcgaaagat ctatgacatc gttatactct tactgcatca gcactgtcct gctccttgtg
8041 atgttacaaa gaccggcttt caaccactca ccatctctcc gaaagacatt attattcctc
8101 cacctaggca cgtagtgagt actcgcgacc cgggggcatg caactgcagt agtcgacctt
8161 aattaagttt gtttattttt ctaaatacat tcaaatatgt atccgctcat gagacaataa
8221 ccctgataaa tgcttcaata atattgaaaa aggaagagta tgagtattca acatttccgt
8281 gtcgccctta ttcccttttt tgcggcattt tgccttcctg tttttgctca cccagaaacg
8341 ctggtgaaag taaaagatgc tgaagatcag ttgggtgcac gagtgggtta catcgaactg
8401 gatctcaaca gcggtaagat ccttgagagt tttcgccccg aagaacgttt tccaatgatg
8461 agcactttta aagttctgct atgtggcgcg gtattatccc gtgttgacgc cgggcaagag
8521 caactcggtc gccgcataca ctattctcag aatgacttgg ttgagtactc accagtcaca
8581 gaaaagcatc ttacggatgg catgacagta agagaattat gcagtgctgc cataaccatg
8641 agtgataaca ctgcggccaa cttacttctg acaacgatcg gaggaccgaa ggagctaacc
8701 gcttttttgc acaacatggg ggatcatgta actcgccttg atcgttggga accggagctg
8761 aatgaagcca taccaaacga cgagcgtgac accacgatgc ctgcagcaat ggcaacaacg
8821 ttgcgcaaac tattaactgg cgaactactt actctagctt cccggcaaca attaatagac
8881 tggatggagg cggataaagt tgcaggacca cttctgcgct cggcccttcc ggctggctgg
8941 tttattgctg ataaatctgg agccggtgag cgtgggtctc gcggtatcat tgcagcactg
9001 gggccagatg gtaagccctc ccgtatcgta gttatctaca cgacggggag tcaggcaact
9061 atggatgaac gaaatagaca gatcgctgag ataggtgcct cactgattaa gcattggtaa
9121 ccatggatcc atggttagcc ctcccacaca taaccaggag gtcagattat gcagtttaag
9181 gtttacacct ataaaagaga gagccgttat cgtctgtttg tggatgtaca gagtgatatt
9241 attgacacgc ccgggcgacg gatggtgatc cccctggcca gtgcacgtct gctgtcagat
9301 aaagtctccc gtgaacttta cccggtggtg catatcgggg atgaaagctg gcgcatgatg
9361 accaccgata tggccagtgt gccggtctcc gttatcgggg aagaagtggc tgatctcagc
9421 caccgcgaaa atgacatcaa aaacgccatt aacctgatgt tctggggaat ataacttaat
9481 taagtctaga tcggatccaa ggtaccctga tcgatatcga gctcttgcta gcagtactga
9541 attcgcattc acattcaata taagtgccac tatcagcaat ctatctatcc cactaactgg
9601 atagaaacat cgttattcag ccatttgtaa gagaagacgc aataggaaca tcaagatcta
9661 tgtcgggtgc ggagaaagag gtaatgaaat ggcacgggat agaacgttat tcaaagggta
9721 atctgcccac ttcaaggaga gttcaacaaa actatgcaga agtcactaaa tgaaccatgc
9781 tgccaaaggc aggcattgga gagaaaacta gaagtagcta aatagtttta attctttcct
9841 gtctacagac acatagattt taacgaagga ataccatagt atagaattga acttttaggc
9901 tgccttctag tcttggttaa atgcatcagg ctgcagtggt aaaattgaat acaacagagc
9961 ccttacagga aagaagtaga tctggatgtg ttttcttggg gagctgttta aaatactgtt
10021 tttgggaaag cacaagtttc agaacagtca ttgtaggcat cgtattcatt gttccattta
10081 tttttacaca cacacacaca cacacacaca cacacactct cacacattgc tatgtgtaca
10141 caaaaataat ttggaagaac ctatacccaa caatttggag tggtcattta tttgggatga
10201 ctggcaattc cctttctatt ctcttcattt ctgcttgttt gtctttaacg agaacgactc
10261 ataatccaaa aatttaaaaa agtataaagt tatctaaata agaaattttc ctctgaagat
10321 gcatcctcag gttggggaga tattaaacaa tgagaaaagg ccccaatctg ggatctgaac
10381 cttgggggag ctgcccatca tttatagaag cacagccttt gggaacaaag caaagtcact
10441 agcaatgtga gacttcctac tcttcatggc ttcatacagt catccatcgc tgttgtgtta
10501 atgaccatga cctgtatgtt agcaggtaaa tgggaaagga agtgggggca aaggagtatg
10561 tgcaggaatg atcaaaataa ggaaaggaag agagggatct ggaaatcacc tgaatgccga
10621 taggtgaaca ggtagaattc ttttaaagct tcccccaccc ggtacccccc aaataacccc
10681 tttccagctt tggaagtttc actaggacat acagtgctca tcctctgatg tcaccttaag
10741 tttggctctt ctggtttgat gagcttgtag cccactagga gctcaaggca tgcatggggc
10801 cacttgccag cacgatgagg ggcatgactg tcatggccaa gtgaacatca aagcagatcc
10861 ccagggctgt atgtctcagg ccttggtgca catcagaatc acttagaaac atccacattc
10921 ctgggccctc ccaccacaaa ctgacagctt catccagggt gtggcccagg catcgggagt
10981 ttttccaaca gctccatggc tgattctcaa cagaaaacca ctggcccaga gcaagggtgg
11041 aggcagcgtg gcatagggct ctgaccttgg ccttgccact gaacctctca gagccccagt
11101 ttctttatgt gtaaaatgag tgtaattata gttcttttct catgaaggtg ctctgactat
11161 taagtgaaac ggggcacatt gtatgacacc taatagctcc tcactaactg gtacccggca
11221 ttataaaggg caggtatgga agggttctgg gagtccaata cccttcttaa agacagagag
11281 gtctctgaga cccagagagg ggcaggcctt acccagagtt gctcagccag agggcaacaa

11341 ggcccaggtc agatgcaggg cccctccacc accactcagc tgcctccaga cccactgcct
11401 tcgccatgtt gttggtagga cactgcatcg cccccacaga aggggcttgc caacttgagt
11461 gagaggactt gcacacttct ttgactttc ttttgagatg cccacaatct gaacaagggc
11521 acttcaaggg acagctctgt caccaaactc atctgaggcc tgaataccat gggtcaggca
11581 ggaatgggtt ggagaggtgt agagcaggca caataagagg gctgaggccc atgcagtcat
11641 cagtgcccac tttcccagga gtctgactgg gcacagcacc catagtgtcc ctgagctggt
11701 ccatggagca gctcactaac tgtttggccc acagcaggtg ctcagtaaat ggcagttgaa
11761 cgaatcaatg gacaaaggaa cataaattac ccaacacaca gggagctcag ccatttactc
11821 aatccattat ggagtaacct acaaacaagc cactgggtcc caaactgaaa ttgtgtctct
11881 tctacattct cccaaagaat ccaataggtt aaaaatagaa atgtatgaaa tagatcaatc
11941 agggatgatt gcatgtggat ttgacataag gatcccctgc agggagtctg agctggcaac
12001 agtcaggccc aaagtgctgt ccatgatgtc tcgaactgca agacagtttt aacaatggcg
12061 aagcaatgca gaaccaggca ggccaaggag ggggtggggg ttggggaaag gaagggaggg
12121 aaggggctgt gaggggcaat ggtctggcat ccctgccacg tgagcctctg aaatttgctg
12181 gcagcttcta tgggctccca gagctttcac ttaattgttg gtctgccact aacctgctgg
12241 gagtaaggtg cagggatgga ggaggcaggg catgaccacc agacactaaa ggtaccagct
12301 ggggccactg gcaaagggaa ggaggctgca cctctcctac atgagagccc gtatacacac
12361 accttttcca gcactcatca actgcatccc aagcaaatgg tccctgatca attccaattc
12421 tagaaaccaa ctgactactc aataacaaag tagatcccag caggccgcca ctgctggagc
12481 ggatgccact tttgctatgc caagtctgtg gctggacagc tgctggcatg tacactcact
12541 gactttcata aggatgccta ataaaggggg caggctcacc tggcttttct caggggtggg
12601 gtttggggtg ccgatagagg ctgctgtttt ggcagagtgg caagctgcaa gcctcttctg
12661 agctttcatt tttcaatgga cttcagtgag aattcacttt gtcagaggcc atgcagctcc
12721 atgttttgga tttcatggaa tgagctttca acagtgagcc tgaagtgccc tggctgaaca
12781 gcaagaacac cagccaaccc taaacaaggc cgaggagagg cggctgtgtt tacacggaag
12841 gctcagcctt gctgtaatag cgtctgcctt caccagacat cagtgaggcg tggaaatcta
12901 ttatccagtt aattttgccc ctagataaag acttgctttc gtgtcttctc tttcacagtc
12961 ccatgatctg ttactcatct caactgcgag aagttggctg ggctttcccc tgtgcccagt
13021 gccacactcg tgccttcact gggtcacctg tgcctgtggc tgatgccgct gaggttttgc
13081 ctgcccagac tgggtgtttc tgactaaatc ccacagccac cattttagat caagggcagg
13141 agatagctca ctgctccgga atgacctccc ctcccagaat cctggtaggg gcggaaggtc
13201 cccaaccaag ctcccagccc tttctaaatg aatctccctg cttcacccat gtgcttttct
13261 ccagtctctg cggtcttgat gacagcaggg tattagtcct agctgtccca cagctcctac
13321 ttctttcagg cctctccctg tgacaatcag tagccactgg caggatttcc tcagagcata
13381 tctcgatttg ctttcagaca attagttaaa aggacactgg accccagacg tcccaactcc
13441 cagccagagc cctcacaggc ccggcctttg gtggtgagga agggggaggg agtgagtgac
13501 agtgccctgg catcttttag aaacgaattc ctttctctcc atacataaat gcctgcagag
13561 tcccatttca gaatccggca gacaaagcca ccaatgtgat ccccatgacc ttataaacat
13621 tcattaaaat gcatttcaag gcatgtgatg gcctccccac cccctagata atgagaaaac
13681 aaaggtttct cttctgatag agacaagttc agctctgaag tcaacattat ttctggttct
13741 gtctgaacaa tgacatatgg caactcttcc ctttctatag ttctagtcca gaatgacaaa
13801 aaaggggaaa aatttcttag agaaggtaga gattatacga atacagtcca tgaaatgagc
13861 ataaggagaa taaagaatat aacttatcca aagaagtctg gcaggctgtt ataaatgctt
13921 gattttggac actgtagttg gaggtttaac atggacacca ataaaaaggt cagcaaaggg
13981 tatgcactgt tcctattggg caagaagata ggaggtcaaa ggtaaccagg aaagataaac
14041 tcagggagac ttattttccc tccagagggc actgggcttg taggccctgg gcaaaattgt
14101 caaaaaggtg aaaatcgcct gtggtttatt tagtctgctc tttcttcact agtgcctcac
14161 cagttcagtt caggccaatt tgctagccag ttctcctagt ctcttccctt acatccatac
14221 tccatgaaca actttattca atattgttac attttccccc tatgaatttt gaggcacctg
14281 atgttggcca actaaattag acttcaagcc ctctgaggac aaggatggcc cccagtagac
14341 tatctcagaa gaaagtaatg aagatgtcgt ggggcatgat ggaggtagct gtagaataga
14401 taagaaaata atgtatcagg aatgggtttg aagagacaaa gagatggggg atggagagaa
14461 agagagagag tgagagcgag gaggagagaa agacagaatg aatatgtttg catttgcaca
14521 tgggatcaaa ggaatatccg tgtgtatagt atcaatgcct gtatttgggg atgtgttatt
14581 tacatgttta ttttcagcag aatgtcccag caaactgaaa ctgatctttc tgtagaacaa
14641 acaggatttt gagcatttcc agaattgcaa gacatgaggc tctggagaat accagggtgg
14701 ttgggaatgg cagggaaccc tgaatgctaa tgctgattgg cttcaagagg agtcagttat

14761 gtctcaggag caaatctctg ataatgggtg gtttggagca agaacctcga cctacaaatc
14821 ggggatttat tggtgtccct gattcgccct gaaccagcac tcgatgcagg cagatgcagg
14881 ctgcggtgga ggaaggggcc ctggaatgca catgagaaga cctgcagtta agtcctagct
14941 cctccgtttg ttgtgtggct gtgggcaagc cactcccctc cctgaagctc aatttcttct
15001 tctgtaaaat gggtacttgg cccaccatgt gtgcttcaca gcattagtga aaggttccac
15061 tgtactcact gaggagagca cttggggagc agtaatcact actgatatca ttctcaaggc
15121 cctgtgtctt tgtgttgggc ctctgttgct ttcttacaaa taaggatgca ttcactagga
15181 tagggggatg gagtggaagt agggggaggc aagcagctta gtcctgacag cactgacctg
15241 acagcactga cctgacagcc agggatgctg tcttggtggc tgagcctgga aactcctgcc
15301 tcctgctgct tgccaccacc tgccactctg aaggctgcca cagtcatcag cctttagcca
15361 ccctgacctg agggctgggg cttcaacagg cttctgccag aactatctga gcagcagcct
15421 ggagtggctg gaagtgcact gacaaaggca gagatgagca aacctgggtg ggcagtgttc
15481 tagaactact ttctcctttt ctgtagtgta gacatgccag ttggaagaac tgacagctct
15541 aaggatgatc ctcaacctat agatgtatat gaatttaatg agctcagaat ttgtattgta
15601 ccatctcctg agaagaatca aggtattctg ccattgaaca agcatttgtt gagagctagc
15661 ctctaactcc tagaccgtca gaactgctgg gcccttcaag acgggctgct cacacccact
15721 catgttaagc ctggtgaggc ctgtactctg ttttcacagg aagaaatcct cacccagtct
15781 tccccaaaca cattcccagg ttctgtcatt agtgggatag agatgattac tgtggggaga
15841 agagaaacat ctggatggat ttggtgaggt tgatctatag aggaagtagg tgctgcctga
15901 ggtagctgta atagaagcta aaggtcaaag gagagggccc tgtcccaatc cagatgactc
15961 cacttctgct ggacccaggt tcacaagctt aatctacatt tcacctaaat ttggctaaca
16021 agcccaaaat cacacaggca aagggagaag tggaggcaga accgaggttg gaggccacca
16081 gggccaccgg gcagagatca tttaagccca accttctcac ttctccctgg gctctgcctc
16141 tcttaaagga ccttgtggtg tgacctcttg taggtccctt tcacactcgg ggcctcagtt
16201 tccccactgt aaagtgaatg ggtcccagct ttggtaagct tatgcttacc tgatgctttc
16261 ttcctgggct gctcttgtag agaaaagata aatcttcttc ctccatccac gagggcttct
16321 ttccctgggg gtgagagtag gctgaggaga gccacttgca cacactctta aagaaagtat
16381 tacctgcacc agctcagtga gaggcacaga tcagactgtt acttgaatca aattatgagc
16441 ctccccaaat atatctatga catttaaata ggggattact tgaacataga ctttgggatc
16501 cggtgtggag tgcaggagac tagcaaagtg aatcctgaga gtagcaggtc tgcacctgtt
16561 ggatcgagaa aggcggccta caattctggt caaatgagct gtgcttattg acatattcta
16621 ttagagagta ctaccaggtc accagtcacc agaaaggctg ccagctctcc aaccacctcc
16681 agggaactat cctgaatggg gccttaacaa gcctaagaga gggttggttt gggtcccaag
16741 ccaatatttg ctctgcttta tgtcagtcat atggaaccca aaccaaccct ctcctatgtg
16801 cctcaccagt cggtgcaggg atcccaattt caagtttggt tttttatggt caaagtccag
16861 catagattaa atgaaggggt gtgatgatgg tgttaaaaga gaactccaga ccagtttaac
16921 tcttggacac acatcccatc tcaccatggt gcttccaacc ttccagagat gatgggctcc
16981 tattttctga tgacaaagcc ctccacagga ttgctgcctg gccatcaggg agtgcctctg
17041 taactgaggc tgagatccca ctttcagtcc tccagctgtg gcccatccct gctccgccca
17101 ccgggtatgg cctgtcctag gctcttaggt atggctgcat tgtgaaatga tggctacaga
17161 gctggcatct cctgtagtct ggttcatcta gtgcactacc tcatagttaa aagaaatctg
17221 tttaagccac tgagggtggc tcctagtgcc aactccaaga acaggaagct tcccttttttt
17281 gggaggaggg gcagatggta acatggatcg tccaggtcaa tgggagcagg gcaaccacag
17341 taagtactgg acaacaacac aaaactccat gtgtggcttc catcgagtcc ctctccaatt
17401 ggtttggtct tctccgtccc atgcagcact ttagcaaggg gcctggctga aggctatgaa
17461 ttgtgtggag cctcctcatt gcagtctcca accatctgat gctgggaaaa tgtcaccagg
17521 atgcagccat gccgtgtggc caatgaaccg agaaaacacc ccttttctag aatgctctaa
17581 agaggcagaa taatccagag gtgaggaagg aaatactcca ccagagaccc aggcagttcc
17641 tacaaaagcc agactttcct tcacctaggg agtgacaaga ccagtggaaa acactctcaa
17701 gcagtaaccc ccaaatgctc tgcaagccag tggcgtccag ataccgcaca agcgagtggg
17761 ctgtctaatc ccatcatcat gatgtaaata tctctaggct gccctgggct gtgcctgacc
17821 ctgtcttcag ctttccacac ctccacctac agcccatgca cagaaggacc acccaggaat
17881 gctgcaagtg tggcacctcc agggccaccc agggagaagg agggcagcta tgctggtggc
17941 tccaggccca tttggcgggt ggtaccttca caccacaaag cccaaactga ggccccagat
18001 ttggctgatg agggcatatt ggacaggggt cacttatgct cttccccatt gccacctggc
18061 ctctggctac ctggacttgg ctacctgtgg atcctctcac aggtgccacc atcttggctg
18121 agtctccaga tgcgaggtcc ctgaggcagt ggcaggcttc tcgctaatgc tgatgggatt

18181 aggaatggga taggtgggga gggccctgga ctgggccctg atgagccaag tgggttttta
18241 gaggggctac tggtacattt cagggacagg acatctggta gagctaagct ggggcaataa
18301 ggagccactg ctaatctgag agctagaaac aatcagcttc tgggtcatta ttaattaggg
18361 tagtttgggc tgtgtggaag tcacgtacta tatggggtag ccacagctct ctctacagat
18421 aatctctaag acttctgatt gggaccgtgt gaatgcagta gcaatatctc ttcttactgc
18481 caggccctgc cagtcctgcc tccacgccct ggctggcccc ccttatgatc tgacccatgc
18541 caggctgcca tagtatgtta cttctgcatt agcactcctt gggacctgcc tctccactgt
18601 ccctcagact ttaaagaact atacaaaccc aaggggctct tcccaagaga attgatatga
18661 cttgaggtga ttccatttct ggaagtagtc actccatttt ctgcctcact ctttcagtgc
18721 ttcacagagc aggttcgcgt atacatcggt gaaccatcac cctaatcaag tttttgggg
18781 tcgaggtgcc gtaaagcact aaatcggaac cctaaaggga gcccccgatt tagagcttga
18841 cggggaaagc cggcgaacgt ggcgagaaag gaagggaaga aagcgaaagg agcgggcgct
18901 agggcgctgg caagtgtagc ggtcacgctg cgcgtaacca ccacacccgc cgcgcttaat
18961 gcgccgctac agggcgcgtg gggatacccc ctagagcccc agctggttct ttccgcctca
19021 gaagccatag agcccaccgc atccccagca tgcctgctat tgtcttccca atcctccccc
19081 ttgctgtcct gccccacccc accccccaga atagaatgac acctactcag acaatgcgat
19141 gcaatttcct cattttatta ggaaaggaca gtgggagtgg caccttccag ggtcaaggaa
19201 ggcacggggg aggggcaaac aacagatggc tggcaactag aaggcacagt cgaggctgat
19261 cagcgggttt aaacgggccc tctagactcg agcggccgct ttacttgtac agctcgtcca
19321 tgccgagagt gatcccggcg gcggtcacga actccagcag gaccatgtga tcgcgcttct
19381 cgttggggtc tttgctcagg gcggactggg tgctcaggta gtggttgtcg ggcagcagca
19441 cggggccgtc gccgatgggg gtgttctgct ggtagtggtc ggcgagctgc acgctgccgt
19501 cctcgatgtt gtggcggatc ttgaagttca ccttgatgcc gttcttctgc ttgtcggcca
19561 tgatatagac gttgtggctg ttgtagttgt actccagctt gtgccccagg atgttgccgt
19621 cctccttgaa gtcgatgccc ttcagctcga tgcggttcac cagggtgtcg ccctcgaact
19681 tcacctcggc gcgggtcttg tagttgccgt cgtccttgaa gaagatggtg cgctcctgga
19741 cgtagccttc gggcatggcg gacttgaaga agtcgtgctg cttcatgtgg tcggggtagc
19801 ggctgaagca ctgcacgccg taggtcaggg tggtcacgag ggtgggccag ggcacgggca
19861 gcttgccggt ggtgcagatg aacttcaggg tcagcttgcc gtaggtggca tcgccctcgc
19921 cctcgccgga cacgctgaac ttgtggccgt ttacgtcgcc gtccagctcg accaggatgg
19981 gcaccacccc ggtgaacagc tcctcgccct tgctcaccat ggtggcgacc ggtggatccg
20041 agctcggtac caagcttaag tttaaacgct agccagcttg ggtctcccta tagtgagtcg
20101 tattaatttc gataagccag taagcagtgg gttctctagt tagccagaga gctctgctta
20161 tatagacctc ccaccgtaca cgcctaccgc ccatttgcgt caatggggcg gagttgttac
20221 gacattttgg aaagtcccgt tgattttggt gccaaaacaa actcccattg acgtcaatgg
20281 ggtggagact tggaaatccc cgtgagtcaa accgctatcc acgcccattg atgtactgcc
20341 aaaaccgcat caccatggta atagcgatga ctaatacgta gatgtactgc caagtaggaa
20401 agtcccataa ggtcatgtac tgggcataat gccaggcggg ccatttaccg tcattgacgt
20461 caatagggggg cgtacttggc atatgataca cttgatgtac tgccaagtgg gcagtttacc
20521 gtaaatactc cacccattga cgtcaatgga aagtccctat tggcgttact atgggaacat
20581 acgtcattat tgacgtcaat gggcgggggt cgttgggcgg tcagccaggc gggccattta
20641 ccgtaagtta tgtaacgcgg aactccatat atgggctatg aactaatgac cccgtaattg
20701 attactatta ataactagtc aataatcaat gtcaacgcgt atatctggcc cgtacatcgc
20761 gaagcagcgc aaaacgccta accctaagca gattcttcat gcaattcgat agatctcgaa
20821 cgaaggagcc atccaactaa ccgtcatgtt cgggcaaccg aagaagggag tggcaggatt
20881 tcctttggag acttctggaa ttagacagca gtttaatgca agcatctaaa ttctcttcct
20941 cccagagtct cattaaaact acagtaagag tttgtgtttt gttttgtttt taaagacaaa
21001 atcccaccag gatagagaga ataggagagg agataacagc atcataattt atgaaactaa
21061 aatgcagata gaccaggatt aactgactac acagcaccaa ggaagctgaa tcacaagaca
21121 gcagaggaga aaactggaaa ggatcgtggt ctatacggca gaatcttccc aagcctcagg
21181 aggaggagct ctagatgttc ccagatctgg gaggtaaagt ggaatggggg gacatggtca
21241 gcgtaatggg gttgggctgg aagctggtta aggagcaggc agatctctga atcccctctc
21301 tgactctgtg tccccaggca tctgcctgtc ccccaccctg gaagaggtct ggcttgaccc
21361 tttgtctggt gaatttcctg ctctgctttc ctggtcctgc tggccagatc agtggaggcc
21421 actcacttca ccccacaggg atgttctgtg ttgccctaca cctgggaact ggaggtactg
21481 gaggcaggct gtggtgagct tgaaagcaaa acacagaggg cagtccaatc tctttggcca
21541 tatttcttct gcatatccaa taccatgtcc acaactctgc tagtgtcctg atggtggtgg

21601 gctctacaca ttcccgggaa gctgaaggca gataatgacc aggacaggtc aacctctctt
21661 cttctgaaag ccttcatcta ctaatggcct gggactcttc ccttaaatgc ttagattgtg
21721 tcttccacta aggttttttg ctgttgctgt tgtttgtttg tttgtttgtt tgtttgtttg
21781 ttttgagacg gaatctcact ctgtcgccca ggctggagtg tagtggcaca atctcagctc
21841 accacaacct tcacctccta ggttgaaggg attctcctgc ctcagcctcc tgagtagcta
21901 ggattacagg cacatgccac catgcctggc taatttttgt atttttagta gagacaggat
21961 ttcgccatgt tggccaggct ggtcttgaac tcctgacctc aggtgatctg cctaccttgg
22021 tctcccaaag tgctgggatt actggtgtga gccaccacac ccggccaagg tttttgtttg
22081 tttgtttgtt tgtttgtttt gtattgaggc agggtatcac tctggtcacc caggctggag
22141 tgcagtagtg caatcacggc tcactgaaac ctccacctcc ctggcgggct caggtgatcc
22201 tgccacctca gcttcccagg tagctgggac tacaggcttg taccaccact cccagctaat
22261 ttttgcgttt ttagtagaga cagggtttcc ccatgttgcc caggttggtc tcaaactctg
22321 ggctcaagcg atctgcctgc ctcagcctcc caaagtgctg ggattacagg tgtaagccac
22381 cgtacccggc cccgccacta aggttttgaa aatgaagcaa ttacaagttt aagtctatta
22441 ataagtgatg aagctatgta gaaaagcaga ataattatct tggatcagga aggtcacatg
22501 aggatctact tgggggttgt caatattcta tttcttgacc tgatcagtgt tgacagcagg
22561 ttttaatttt ttacttcttt ttgtttgttt gtttttgaga cggagtcttg ctctgtctcc
22621 caggctggag tgcagtggta tgatctcggc tcactgcaac ctccgcctcc tgggttcaag
22681 ctgttctcct gcctcagcct ccccagtagc tgggattaca ggcaggcacc accacgacca
22741 gctaattttt gtatttttag tagagactgg gtttcaccat cttggccagg ctggtctcga
22801 acttctgatc tcgtgatccg ccctccttgg cctcccaaag tgctgggatt acaggcttga
22861 gccagcgtgc ccggcccatt ttttacttcc ttattaaact gtacatatag gccttgcaca
22921 cttttctgca tcaatgttat attccacaat aaagggaaaa ggtatataca caacttgata
22981 ccagtaatgt gaaacatata tttctacata gaaaaaaaaa tgactgaaat actgcactcc
23041 aatgtgttca cacagtagtt gtttctggat tatttatata ttaaatgttt atatattgta
23101 ttatgccatg aggtttgtgt tttctctcca cttttctgca ttttccaagt ttactacaaa
23161 gagcacatat tactcttata atcagaaagt cataaaatat atttaaaaag acaaaattga
23221 aactaataag gatcaacaca aaacagatga gccatctgtg gaaatccgca cagaatacta
23281 cctaaagaga ttggtgacgt gcatgatctc actaggatga gcacaaagct tgccagagcc
23341 tagggtctat ttctagggtt ggctcttgga agccaggata gttgttatct ctgggaagag
23401 ggaggggcac acaaggggct tctaaaacat tctgaatgtt ctatttctga acctggttgg
23461 tgggtacatg actgttggtt ttattattat atgttttata tactcttccg tatgtatggt
23521 gtggattcca aaaaaagatt tcctttagag aaaaccagaa tcacataagt agaaaatatg
23581 gtgctatgtt gaaggaacaa ctcaagttta tataaaatca tcatcattta taggcttaaa
23641 aagttgcttt ggaattttgg tctaactgac ttgtcttttc tgcagcaaac cacgctcctt
23701 ctggacgtgc tccaggcaga ggggattagg gtgggttcaa ggctgcaagt acctagctca
23761 gcacactctc ttcaggggac ttagagtttg tctggtgttg gctctctgag ctcttgtcag
23821 gaatgccgac ccttccgagg ttcaggattt gaagcctgcc ttcccacccc agatttggtc
23881 cacacagaca ctcaagtatg tatttcaact acaaatgacc tgtactttcc tattactcct
23941 ctctttcatg gtaacctttc tggtatcctt ccttccctac atttatggga gggggacatc
24001 attctctgct ctcctgtcac tgaaggctcc accttctgtc ttcttctgac ccatctggtt
24061 ttcctggggc cacctcctct ccttaccacc ctaacgcttt tgtaacttga ggagaaatga
24121 gagatcacct agtcaggtca tcattctctg tagatgaaga ggcccaatgg tttgctcaag
24181 aattgccaag cgagttaaag acagagagta tgagagtcag caagacctac agaaagcatc
24241 tatctgcact gttttgcagg gacttagcct ttgtgtgtgg actcctggaa tgccacccac
24301 taagaaacat tgtctgacac caactcccca cttggtaggt ggggacactg aaactcatgg
24361 caggaaaggg ccttgcccca agccagggca gagtgtcact catcactctc aattttcagt
24421 ccagggcacc ttgttgtgac tatcccaaag gcagccactt tccctggtct gaaagacctg
24481 aagagagaag agaagagaag gatggaaggc agagtatgcg gctttgattc atttcctggt
24541 gaaaacagat ctatacgaga agcaaatttc acgaaaggga agagaagaaa gtgtcccata
24601 cgttgctggc ctgtttcaac cttgctttga ttcttgctga aaagggtacc gtgtatttct
24661 gagttcaaca tgcagaccag tgttaggaaa gccactgcac ctccacttta gcctccaggg
24721 ctgtgccctg caaatggcct gcagccttgg tgcctcgctc tccagactgc attttggaag
24781 atgggacaga ggcttatgga agcccacatt agaacggggg agcagaatgg gtgagatgag
24841 ggatccttga tagtgaacca gatgaaggaa tggtagccaa atgccaggcc tcctttgtgg
24901 cttcaatcca aaggctctgg agcccttcca gggcagaaca tcaggcatgt ttacccccac
24961 tgtcctcaac agtgacagag gtgcaatctt gggcagctgg ccattttgaa agcaacctcc

25021 ttaatctcaa ctgggaaggc tccctagcag gacccctgtg ttgcacacct ggaggaagct
25081 agactaacca gaagctcagc acggttccat ctgggatgcc caggtctgag acgaaaaagg
25141 taactctctt ttctgggtcc tggcccagtt gtgtctctct ccacctcatt ctctgagatg
25201 cctgtctccc ctttttgtc ccatcaggag gcaagagcta tcactgggcc agactccacc
25261 agaagccaag ccagcttgtt acccagcttc tcagggagca aagaacagcc ttgtttctat
25321 cttatcccca ctgtcccctg cccctgcccc acctcccagc cattcagctt ctggcttccc
25381 cagagctgcc tgcttctttg tggtcctcca ttccttgaaa agaccttcta gtcattagtg
25441 tatataaatg gccacttagc ccagattaca gtgaggtcaa cagctggggc tctgagaatt
25501 gtcacacact ggcacaggag aggaggctat tcttccagag aatttggagg gcactcccat
25561 ccacttacaa caaaaagccc atccactgtg cttggcagta ggtgatctga gaaccaatgg
25621 aaccaggtta atcctgtggc actgttgagt gaggagagca gtggcgggca ctggaaaata
25681 tcagagacaa ggcaggagac ctgaaatcta ggcttagctc ctcatatact tggcagctgt
25741 atgacctcag acaaccagtg ttacctctct aagcctcagt ttcctcatgc aaaaggaggg
25801 ggaataacaa cagagcccac tgcttggggg tgttgtgagg acaggatgaa aaaacaaaca
25861 gaaatccctc agtacaggat tcagtgcagt ggacagtctt gcaaggtctg gttcagccct
25921 ccacccctac cctcaccagt ataaagaact ctggcctaca agtcagatga cctgagtttt
25981 aatctcagct ttgccattag ccgtgtgaac ttgagaaagt ccctttcctt tttacatcta
26041 ttgggatgat catgcatttt ttgtccttta ttctgttaat atagtgtgtt acattgattg
26101 cttttcatag actgaaccag ccttgtattc cagggataaa tctcacttgg tcatggtgta
26161 taatccttta tacaaatgtt gctgggttga gtttgctagt attttgttga agatttttat
26221 gtcttgattc ataaggaata ttggtgtacc ttcccctttt atggccacag tttccctaca
26281 atgatgtagt cgaactagac aacctccaat atctttcagt attcatgtcc tctgattctg
26341 tgaaactaag aaaattaaga aatagtgatt cataggcaca aggcaggcaa aacttagact
26401 ccttgtagaa taattaggaa gccaaatatt cagtgtgctt atttctcaaa taaccttagt
26461 ttctccagtc tgccccaact ccgaggcctg aatatctcta gatgcttatg atggcaacta
26521 aagcctaaaa gctaattcat tttaaagttc ttccaaatgc atagggtttt atttttccag
26581 acctgggttc agatggggaa tttgacaaac aatggaaagg gggaaaaaca acaatctaaa
26641 cactgagtga caaagtaaca aagaaatagt ctagctatca gccagtcaag ccagccttgg
26701 ctttgctatc caaagtagtc agtctaattc taccaccagt ttctgttcct gtagctgtct
26761 actgcctgcc agggactctg ccttcccacc cacaactacc aatggaagga tgtggtgacc
26821 ataccagtgg ctgctgacat ctcctgccat gggaagcata attgcctcca gcagcctccc
26881 ccttagatcc atcattttg ttgcacttgg cctgggctgt actcccggcc aatgactgaa
26941 catggtgagc atagtaatgc aggcccattt ctgtgaggag caggactcct ccagtaggtg
27001 actttggctc aaggactctc tattggcctg gttgaacttt tcctgaactg tgctactgtc
27061 tgagactctt cttacccaat cctctttctc gccccaattg tcacagacca cctgcattgt
27121 ggtctgagtc tctccccacc ttctcttgct cttccctgtt tatctttcac aggcatttcc
27181 cccagtacat tccttgaatg tctaacccga tacgggtgcc tgacttttgg cagacctaag
27241 cagacaaaaa ggagtacttg gttacctagc tcttctttct accacaaaca tcgagggaac
27301 cctttttccc tcacccctct gccacacccc cactgcccca gtgaacaacc acagagagag
27361 ctgtggtata atattaggct ggtgcaaaag taattgcggt ttttgccatt acttttaatg
27421 gtaaaaaccg caattacttt tgcacctacc tagtatttgt gtccccccaa attcatatgt
27481 tgaaacctaa cccacaatat gatgtcatta ggaggcaaga ccttgaggag gtgattagat
27541 gatggggtgg agctctcctg aatgagatta gtgcccttat aagaagaagc ccaaggaagc
27601 taccttgact cttccatcac atgagaatgc agcaagaagg caccatctac taatcaggaa
27661 gagagctctc accagacact gaatctgcca gtgtcttgat cttgaagttc ccagcctcca
27721 gaactatgca taatgcattt ccattgtctc taagccaccc agcctatggt attttgtcat
27781 agcagcctga actgactaag acagtgagcc acatgagaag tgccccaacc cctcccttaa
27841 gcacttggct cacagatcag tgggttcatt tctgcctgag ttttattgtt attctgtaga
27901 tttcttgggc tagatatatt tttctgttat tttccttctt cacctcagtc atgaattggt
27961 tgttttaaaa aagacaatgt aagtcatggg gaaactcctg acaactctac tctcctaggg
28021 ttcctgataa aaggggattc agttgagtcc tctgatggtc tctacctgcc aaagtccagc
28081 agcccttagc aaacatgctg ctcgtttctg tagagaaggt gctggtgtcc caccatactt
28141 ctctctccct catgaagggc ttgcgaccca gcaaatgggt ggcttatatg ggtctgtttc
28201 aaaggaagag ccagctctgg gaagaaaaac gatgagcata agcataacct accactgtgc
28261 ctgggaaagc agacaacttt tttgatgtgt gaatatctaa tgagaatgga atccatcaat
28321 taccttaaac ttaggcacag tcttcaaatt caatatatgt gggatatact tttagtcagt
28381 ttgtagacgt tatttgtaat aaataatctg gcttctctaa agaaattatt ttaagtgttt

28441 ggtttggttt gatttaatgg taaaattata tttagtggca gagaattata gcaatggtga
28501 taaactatag agtgtcataa gttcatatct tattctcaca tttgaagctg cctgcagatg
28561 cattcaagat gcagccagaa gtcaggagac tcaggctgtt atttggagct catcatttta
28621 cagccttgct ggactcccac tttctcaggg gaaaaatgtg gtgttgaccc agattagctc
28681 tccaggccct gctgagttgg gcactctgta agctggaggg tcttctattg tcttcaccta
28741 agtgtcaatc aacaacccaa atgggcatgg gggaagaggg agctgggcca atgcccaggg
28801 tgcctggtag agagatacct tgggcactgg aaggcaccag cttcccagag agaaggggga
28861 gggccatgaa aaagttggct gtagatgcca gggacactgg gactctccag ctgtgtgttt
28921 gtgtcttctg aagacttatg tttcattcct ttggagcatg cataatcata cactgtggga
28981 tgtgttatat agattgcttg atagttcacc actgtaataa aatactgtga ctggaatctg
29041 ctcccagtct gcctttgata gcacttgtgc aacacacatt tactgagcat ttacagtgat
29101 ccaggacctg tgttgtgaaa acattgatgg acaaggcaga tggtggagca cgtcagtgag
29161 gatttttaac aaaggctggt aagtgctata aaggaacatt gtaggacact agagaacaaa
29221 gaacaggaga acctgactta ggctggggtg gggcgttggt tagaggaggc tccttggagg
29281 acatgaggtt taagctgtga cctgaggatg aatagatgtt ggccaggtga ggttaattaa
29341 gcttgtcagc cttaccagta aaaaagaaaa cctattaaaa aaacaccact cgacacggca
29401 ccagctcaat cagtcacagt gtaaaaaagg gccaagtgca gagcgagtat atataggact
29461 aaaaaatgac gtaacggtta aagtccacaa aaaacaccca gaaaaccgca cgcgaaccta
29521 cgcccagaaa cgaaagccaa aaaacccaca acttcctcaa atcgtcactt ccgttttccc
29581 acgttacgta acttcccatt ttaagaaaac tacaattccc aacacataca agttactccg
29641 ccctaaaacc tacgtcaccc gccccgttcc cacgccccgc gccacgtcac aaactccacc
29701 ccctcattat catattggct tcaatccaaa ataaggtata ttattgatga tgtttaaact
29761 acggcggccg cacgggccat cgatggatcc gcggaagctt ctcgacccat tctcatgttt
29821 gacagcttat catcgaattt ctgccattca tccgcttatt atcacttatt caggcgtagc
29881 aaccaggcgt ttaagggcac caataactgc cttaaaaaaa ttacgccccg ccctgccact
29941 catcgcagta ctgttgtaat tcattaagca ttctgccgac atggaagcca tcacagacgg
30001 catgatgaac ctgaatcgcc agcggcatca gcaccttgtc gccttgcgta taatatttgc
30061 ccatggtgaa aacgggggcg aagaagttgt ccatattggc cacgtttaaa tcaaaactgg
30121 tgaaactcac ccagggattg gctgagacga aaaacatatt ctcaataaac cctttaggga
30181 aataggccag gttttcaccg taacacgcca catcttgcga atatatgtgt agaaactgcc
30241 ggaaatcgtc gtggtattca ctccagagcg atgaaaacgt ttcagtttgc tcatggaaaa
30301 cggtgtaaca agggtgaaca ctatcccata tcaccagctc accgtctttc attgccatac
30361 ggaattccgg atgagcattc atcaggcggg caagaatgtg aataaaggcc ggataaaact
30421 tgtgcttatt tttctttacg gtctttaaaa aggccgtaat atccagctga acggtctggt
30481 tataggtaca ttgagcaact gactgaaatg cctcaaaatg ttctttacga tgccattggg
30541 atatatcaac ggtggtatat ccagtgattt ttttctccat tttagcttcc ttagctcctg
30601 aaaatctcga taactcaaaa aatacgcccg gtagtgatct tatttcatta tggtgaaagt
30661 tggaacctct tacgtgccga tcaacgtctc attttcgcca aaagttggcc cagggcttcc
30721 cggtatcaac agggacacca ggatttattt attctgcgaa gtgatcttcc gtcacaggta
30781 tttattcgcg ataagctcat ggagcggcgt aaccgtcgca caggaaggac agagaaagcg
30841 cggatctggg aagtgacgga cagaacggtc aggacctgga ttggggaggc ggttgccgcc
30901 gctgctgctg acggtgtgac gttctctgtt ccggtcacac cacatacgtt ccgccattcc
30961 tatgcgatgc acatgctgta tgccggtata ccgctgaaag ttctgcaaag cctgatggga
31021 cataagtcca tcagttcaac ggaagtctac acgaaggttt ttgcgctgga tgtggctgcc
31081 cggcaccggg tgcagtttgc gatgccggag tctgatgcgg ttgcgatgct gaaacaatta
31141 tcctgagaat aaatgccttg gcctttatat ggaaatgtgg aactgagtgg atatgctgtt
31201 tttgtctgtt aaacagagaa gctggctgtt atccactgag aagcgaacga aacagtcggg
31261 aaaatctccc attatcgtag agatccgcat tattaatctc aggagcctgt gtagcgttta
31321 taggaagtag tgttctgtca tgatgcctgc aagcggtaac gaaaacgatt tgaatatgcc
31381 ttcaggaaca atagaaatct tcgtgcggtg ttacgttgaa gtggagcgga ttatgtcagc
31441 aatggacaga acaacctaat gaacacagaa ccatgatgtg gtctgtcctt ttacagccag
31501 taggctcgcc gcagtcgagc gacggcgaag ccctcgagtg agcgaggaag caccagggaa
31561 cagcacttat atattctgct tacacacgat gcctgaaaaa acttcccttg gggttatcca
31621 cttatccacg gggatatttt tataattatt ttttttatag tttttagatc ttcttttta
31681 gagcgccttg taggccttta tccatgctgg ttctagagaa ggtgttgtga caaattgccc
31741 tttcagtgtg acaaatcacc ctcaaatgac agtcctgtct gtgacaaatt gcccttaacc
31801 ctgtgacaaa ttgccctcag aagaagctgt tttttcacaa agttatccct gcttattgac

31861 tcttttttat ttagtgtgac aatctaaaaa cttgtcacac ttcacatgga tctgtcatgg
31921 cggaaacagc ggttatcaat cacaagaaac gtaaaaatag cccgcgaatc gtccagtcaa
31981 acgacctcac tgaggcggca tatagtctct cccgggatca aaaacgtatg ctgtatctgt
32041 tcgttgacca gatcagaaaa tctgatggca ccctacagga acatgacggt atctgcgaga
32101 tccatgttgc taaatatgct gaaatattcg gattgacctc tgcggaagcc agtaaggata
32161 tacggcaggc attgaagagt ttcgcgggga aggaagtggt tttttatcgc cctgaagagg
32221 atgccggcga tgaaaaaggc tatgaatctt ttccttggtt tatcaaacgt gcgcacagtc
32281 catccagagg gctttacagt gtacatatca acccatatct cattcccttc tttatcgggt
32341 tacagaaccg gtttacgcag tttcggctta gtgaaacaaa agaaatcacc aatccgtatg
32401 ccatgcgttt atacgaatcc ctgtgtcagt atcgtaagcc ggatggctca ggcatcgtct
32461 ctctgaaaat cgactggatc atagagcgtt accagctgcc tcaaagttac cagcgtatgc
32521 ctgacttccg ccgccgcttc ctgcaggtct gtgttaatga gatcaacagc agaactccaa
32581 tgcgcctctc atacattgag aaaaagaaag gccgccagac gactcatatc gtattttcct
32641 tccgcgatat cacttccatg acgacaggat agtctgaggg ttatctgtca cagatttgag
32701 ggtggttcgt cacatttgtt ctgacctact gagggtaatt tgtcacagtt ttgctgtttc
32761 cttcagcctg catggatttt ctcatacttt ttgaactgta atttttaagg aagccaaatt
32821 tgagggcagt ttgtcacagt tgatttcctt ctctttccct tcgtcatgtg acctgatatc
32881 gggggttagt tcgtcatcat tgatgagggt tgattatcac agtttattac tctgaattgg
32941 ctatccgcgt gtgtacctct acctggagtt tttcccacgg tggatatttc ttcttgcgct
33001 gagcgtaaga gctatctgac agaacagttc ttctttgctt cctcgccagt tcgctcgcta
33061 tgctcggtta cacggctgcg gcgagcgcta gtgataataa gtgactgagg tatgtgctct
33121 tcttatctcc ttttgtagtg ttgctcttat tttaaacaac tttgcggttt tttgatgact
33181 ttgcgatttt gttgttgctt tgcagtaaat tgcaagattt aataaaaaaa cgcaaagcaa
33241 tgattaaagg atgttcagaa tgaaactcat ggaaacactt aaccagtgca taaacgctgg
33301 tcatgaaatg acgaaggcta tcgccattgc acagtttaat gatgacagcc cggaagcgag
33361 gaaaataacc cggcgctgga gaataggtga agcagcggat ttagttgggg tttcttctca
33421 ggctatcaga gatgccgaga aagcagggcg actaccgcac ccggatatgg aaattcgagg
33481 acgggttgag caacgtgttg gttatacaat tgaacaaatt aatcatatgc gtgatgtgtt
33541 tggtacgcga ttgcgacgtg ctgaagacgt atttccaccg gtgatcgggg ttgctgccca
33601 taaaggtggc gtttacaaaa cctcagtttc tgttcatctt gctcaggatc tggctctgaa
33661 ggggctacgt gttttgctcg tggaaggtaa cgacccccag ggaacagcct caatgtatca
33721 cggatgggta ccagatcttc atattcatgc agaagacact ctcctgcctt tctatcttgg
33781 ggaaaaggac gatgtcactt atgcaataaa gcccacttgc tggccggggc ttgacattat
33841 tccttcctgt ctggctctgc accgtattga aactgagtta atgggcaaat ttgatgaagg
33901 taaactgccc accgatccac acctgatgct ccgactggcc attgaaactg ttgctcatga
33961 ctatgatgtc atagttattg acagcgcgcc taacctgggt atcggcacga ttaatgtcgt
34021 atgtgctgct gatgtgctga ttgttcccac gcctgctgag ttgtttgact acacctccgc
34081 actgcagttt ttcgatatgc ttcgtgatct gctcaagaac gttgatctta aagggttcga
34141 gcctgatgta cgtattttgc ttaccaaata cagcaatagt aatggctctc agtccccgtg
34201 gatggaggag caaattcggg atgcctgggg aagcatggtt ctaaaaaatg ttgtacgtga
34261 aacggatgaa gttggtaaag gtcagatccg gatgagaact gtttttgaac aggccattga
34321 tcaacgctct tcaactggtg cctggagaaa tgctctttct atttgggaac ctgtctgcaa
34381 tgaaattttc gatcgtctga ttaaaccacg ctgggagatt agataatgaa gcgtgcgcct
34441 gttattccaa aacatacgct caatactcaa ccggttgaag atacttcgtt atcgacacca
34501 gctgccccga tggtggattc gttaattgcg cgcgtaggag taatggctcg cggtaatgcc
34561 attactttgc ctgtatgtgg tcgggatgtg aagtttactc ttgaagtgct ccggggtgat
34621 agtgttgaga agacctctcg ggtatggtca ggtaatgaac gtgaccagga gctgcttact
34681 gaggacgcac tggatgatct catcccttct tttctactga ctggtcaaca gacaccggcg
34741 ttcggtcgaa gagtatctgg tgtcatagaa attgccgatg ggagtcgccg tcgtaaagct
34801 gctgcactta ccgaaagtga ttatcgtgtt ctggttggcg agctggatga tgagcagatg
34861 gctgcattat ccagattggg taacgattat cgcccaacaa gtgcttatga acgtggtcag
34921 cgttatgcaa gccgattgca gaatgaattt gctggaaata tttctgcgct ggctgatgcg
34981 gaaaatattt cacgtaagat tattacccgc tgtatcaaca ccgccaaatt gcctaaatca
35041 gttgttgctc ttttttctca ccccggtgaa ctatctgccc ggtcaggtga tgcacttcaa
35101 aaagccttta cagataaaga ggaattactt aagcagcagg catctaacct tcatgagcag
35161 aaaaaagctg ggggtgatatt tgaagctgaa gaagttatca ctcttttaac ttctgtgctt
35221 aaaacgtcat ctgcatcaag aactagttta agctcacgac atcagtttgc tcctggagcg

35281 acagtattgt ataagggcga taaaatggtg cttaacctgg acaggtctcg tgttccaact
35341 gagtgtatag agaaaattga ggccattctt aaggaacttg aaaagccagc accctgatgc
35401 gaccacgttt tagtctacgt ttatctgtct ttacttaatg tcctttgtta caggccagaa
35461 agcataactg gcctgaatat tctctctggg cccactgttc cacttgtatc gtcggtctga
35521 taatcagact gggaccacgg tcccactcgt atcgtcggtc tgattattag tctgggacca
35581 cggtcccact cgtatcgtcg gtctgattat tagtctggga ccacggtccc actcgtatcg
35641 tcggtctgat aatcagactg ggaccacggt cccactcgta tcgtcggtct gattattagt
35701 ctgggaccat ggtcccactc gtatcgtcgg tctgattatt agtctgggac cacggtccca
35761 ctcgtatcgt cggtctgatt attagtctgg aaccacggtc ccactcgtat cgtcggtctg
35821 attattagtc tgggaccacg gtcccactcg tatcgtcggt ctgattatta gtctgggacc
35881 acgatcccac tcgtgttgtc ggtctgatta tcggtctggg accacggtcc cacttgtatt
35941 gtcgatcaga ctatcagcgt gagactacga ttccatcaat gcctgtcaag ggcaagtatt
36001 gacatgtcgt cgtaacctgt agaacggagt aacctcggtg tgcggttgta tgcctgctgt
36061 ggattgctgc tgtgtcctgc ttatccacaa cattttgcgc acggttatgt ggacaaaata
36121 cctggttacc caggccgtgc cggcacgtta accgggctgc atccgatgca agtgtgtcgc
36181 gtaatacgac tcactatagg gcgaattgga gctccactac gtagtttaaa catcatcaat
36241 aatatacctt attttggatt gaagccaata tgataatgag ggggtggagt ttgtgacgtg
36301 gcgcggggcg tgggaacggg gcgggtgacg tagtagtgtg gcggaagtgt gatgttgcaa
36361 gtgtggcgga acacatgtaa gcgacggatg tggcaaaagt gacgtttttg gtgtgcgccg
36421 gtgtacacag gaagtgacaa ttttcgcgcg gttttaggcg gatgttgtag taaatttggg
36481 cgtaaccgag taagatttgg ccattttcgc gggaaaactg aataagagga agtgaaatct
36541 gaataatttt gtgttactca tagcgcgtaa tatttgtcta gggccgcggg gactttgacc
36601 gtttacgtgg agactcgccc aggtgttttt ctcaggtgtt ttccgcgttc cgggtcaaag
36661 ttggcgtttt gattc//

References

1. Kennedy, E.M., Kornepati, A.V., Goldstein, M., Bogerd, H.P., Poling, B.C., Whisnant, A.W., Kastan, M.B. and Cullen, B.R. (2014) Inactivation of the human papillomavirus E6 or E7 gene in cervical carcinoma cells by using a bacterial CRISPR/Cas RNA-guided endonuclease. *Journal of virology*, **88**, 11965-11972.
2. Ousterout, D.G., Kabadi, A.M., Thakore, P.I., Majoros, W.H., Reddy, T.E. and Gersbach, C.A. (2015) Multiplex CRISPR/Cas9-based genome editing for correction of dystrophin mutations that cause Duchenne muscular dystrophy. *Nature communications*, **6**, 6244.
